# Maternal weight status and the composition of the human milk microbiome: A scoping review

**DOI:** 10.1371/journal.pone.0274950

**Published:** 2022-10-03

**Authors:** Katherine Daiy, Victoria Harries, Kate Nyhan, Urszula M. Marcinkowska

**Affiliations:** 1 Department of Anthropology, Yale University, New Haven, CT, United States of America; 2 Cushing/Whitney Medical Library, Yale University, New Haven, CT, United States of America; 3 Department of Public Health, Jagiellonian University, Kraków, Poland; Arizona State University, UNITED STATES

## Abstract

The human milk microbiome is thought to partly contribute to the assembly of the infant gut microbiome, a microbial community with important implications for infant health and development. While obesity has well-established links with the adult gut microbiome, less is known about how it affects the human milk microbiome. In this scoping review, we synthesize the current literature on the microbial composition of human milk by maternal weight status, defined broadly as BMI (prepregnancy and postpartum) and gestational weight gain (GWG). This study followed the *a priori* protocol published in Prospero (registration #: CRD42020165633). We searched the following databases for studies reporting maternal weight status and a characterization of milk microbiota through culture-dependent and culture-independent methods: MEDLINE, Embase, Web of Science, CINAHL, and Scopus. After screening 6,365 studies, we found 20 longitudinal and cross-sectional studies investigating associations between maternal weight status and the composition of the milk microbiome. While some studies reported no associations, many others reported that women with a pre-pregnancy or postpartum BMI characterized as overweight or obese, or with excessive GWG, had higher abundances of the genus *Staphylococcus*, lower *Bifidobacterium* abundance, and lower alpha diversity (within-sample diversity). This review suggests that maternal weight status is minorly associated with the composition of the milk microbiome in various ways. We offer potential explanations for these findings, as well as suggestions for future research.

## Introduction

Obesity—a global epidemic with far-reaching implications for maternal and child health—is characterized by excess adiposity, increased energy intake, reduced energy expenditure, and systemic low-grade inflammation [1]. Recent research has implicated the gut microbiome as a key mediator in the pathophysiology of obesity [2]. In particular, individuals with obesity harbor gut microbiota with low diversity and greater metagenomic capacity for dietary energy harvest [3].

Maternal nutritional status may contribute to intergenerational cycles of obesity via microbiome-related pathways. Maternal obesity and gestational weight gain (GWG) are associated with increased risk for childhood obesity and metabolic syndrome [4, 5]. Additionally, obesity in pregnant women is associated with alterations in maternal gut microbiome communities. For instance, one study reported that pregnant women with a prepregnancy body mass index (BMI) of overweight (defined as 25 to <30 kg/m^2^) or obese (defined as >30 kg/m^2^) have higher abundances of *Bacteroides* and *Staphylococcus* in the gut microbiome compared to women with normal BMI (defined as <24.9 kg/m^2^) [6]. This study also reported that excessive GWG–defined as greater than 16.0 kg for women with normal weight or 11.5 kg for women with overweight or obesity [7]–is associated with increased levels of *Bacteroides* in the maternal gut [6]. Another study found that women with overweight prepregnancy BMI also exhibit increased *Staphylococcus*, *Escherichia coli*, and lower *Bifidobacterium* than women with normal weight, compositional features that are typically observed in non-pregnant individuals with obesity [8]; however, this study [8] found that women with overweight prepregnancy BMI had lower *Bacteroides*, contrasting to other work [6]. While this evidence suggests that maternal weight status influences the maternal gut microbiome, little is known about how maternal weight status affects other maternal microbiomes involved in the early life maternal-infant microbial exchange, such as the milk microbiome.

The initial colonization and composition of infant gut microbial communities is thought to be critical for immune and metabolic programming, and is associated with infant health outcomes, including overweight and obesity [9]. For instance, greater abundance of *Staphylococcus* and lower *Bifidobacterium* in infancy were associated with increased risk of childhood obesity at 7 years [10]. Another study found that the composition of infant gut microbiome in early infancy and at 2 years of age predicted childhood BMI, and that the taxonomic subset associated most strongly with later childhood BMI overlapped with the gut microbiota of women with overweight BMIs, obesity, and excessive GWG [11]. While many early life factors affect the infant gut microbiome (e.g., delivery mode, antibiotics [12]), breastfeeding is another critical factor, providing infants with a continuous source of microbes and prebiotic factors (i.e., human milk oligosaccharides) that help to seed the infant’s first gut microbiome communities.

Human milk contains a low-biomass community of microorganisms known as the milk microbiome [13], which accounts for a small portion (27%) of infant gut bacteria [14]. Although previously thought to be sterile, research involving culture-dependent approaches (culturing of microbes on selective media) and culture-independent approaches (i.e., next-generation sequencing) have shown that human milk contains viable bacteria [15]. The origin of the human milk microbiome is uncertain–milk microbes may originate from the maternal skin, the infant oral cavity through suckling, breast tissue, and from the maternal gut microbiome through an immunologically-mediated “entero-mammary” pathway in late pregnancy [16–18]. Milk microbiome composition can be measured in terms of the relative abundance of different microbial taxa, as well as by its alpha diversity, or the diversity of taxa within samples. Previous systematic and scoping reviews have identified a broad range of factors that influence the composition of the milk microbiome [19–22]. However, to the best of our knowledge, no reviews to date have specifically examined how, and to what extent, maternal weight status is associated with the composition of milk microbial communities. As the human milk microbiome is a small, yet potentially important contributor to the assembly of an infant’s first gut microbiome, delineating how maternal weight status influences its composition is a key step in understanding the maternal factors that contribute to intergenerational cycles of obesity.

Given that the milk microbiome is an emerging area of research, a scoping review design is optimal for examining the associations between maternal characteristics and the composition of the milk microbiome. In this scoping review, we aimed to investigate the extent and range of knowledge on the association between maternal weight status, broadly defined, and the composition of the milk microbiome.

## Methods

The purpose of a scoping review is to rapidly explore and describe key concepts and evidence, often in underexplored areas of study. Compared to systematic reviews, which gather specific empirical evidence with a narrow and focused research question, scoping reviews are more flexible in the breadth of literature reviewed, thus allowing authors to comprehensively review the “scope” of a topic [23]. Because of the breadth of their research questions, scoping reviews are also suited to synthesizing topics with heterogenous or disparate evidence [24]. Thus, a scoping review is well-suited to exploring relationships with the milk microbiome.

This review follows an a priori protocol deposited in PROSPERO; because of the 2020 SARS-CoV-2 pandemic, it was published by PROSPERO without an official eligibility check. Its registration number is CRD42020165633 and it may be accessed at https://www.crd.york.ac.uk/prospero/display_record.php?ID=CRD42020165633..

Peer-reviewed journal articles describing prospective longitudinal, cross-sectional, cohort, observational, and experimental studies were eligible for inclusion if they measured relationships between maternal weight status and the human milk microbiome. Maternal weight status could be measured as any of the following: GWG (as defined by Institute of Medicine [7]) gestational change in BMI, prepregnancy maternal weight, BMI, or percentage body fat, and/or postpartum maternal weight, BMI, or percentage body fat. For inclusion, the human milk microbiome could be measured by culture-dependent and culture-independent based methods at a single or multiple time points during lactation. Culture-dependent based methods refer to culturing, isolating and characterizing microbial taxa by phenotype and/or genotype (such as through whole genome sequencing). Culture-independent based methods include next generation sequencing techniques (16S ribosomal RNA [rRNA]), amplicon analysis (metataxonomics), qPCR (i.e., real-time PCR), total DNA sequencing (metagenomics), and gel electrophoresis.

Studies were excluded from this scoping review if they included women who were reported smokers, had a sample of women of whom the majority (>50%) had gestational diabetes, or included women with mastitis as these are known to impact human milk composition, and thus, may affect the milk microbiome [19–21, 25]. Non-human studies and studies in languages other than English were also excluded. No infant characteristics (e.g., gestational age, age at sample collection, birth mode) were included in the inclusion or exclusion criteria.

To identify potentially relevant articles, five bibliographic databases were searched, covering all studies published prior to February 24, 2022 (which includes an original search up to February 13, 2020, and an updated search up to February 24, 2022). These databases were MEDLINE, Embase, Web of Science, CINAHL, and Scopus. Details of database platforms are presented in **S1 Table**.

Each database was searched from inception (without date limits). No records were excluded from the search results before screening because of publication type. Relevant conference papers were identified in the screening process. The conference papers themselves were not included, because through search updates, we identified related publications with fuller reporting.

The MEDLINE and other search strategies were drafted by an experienced librarian (KN) in collaboration with the other authors and was peer reviewed by an independent medical librarian using the PRESS Guidelines [26]. The MEDLINE search as peer reviewed and conducted in 2020 is presented in **S2A Table**; the 2022 database search updates for all five bibliographic databases are presented in **S2B–S2F Table**. The MEDLINE search was translated into appropriate syntax and controlled vocabulary (if available) in the other databases. All the searches shared the same structure: queries retrieving papers about breastmilk, queries retrieving papers about the microbiome (including culture dependent based methods and culture independent based methods), and queries about maternal BMI.

Shortly before submission, forward citation chaining was conducted via Citation Chaser to maximize retrieval of relevant papers [27].

**Fig 1:**
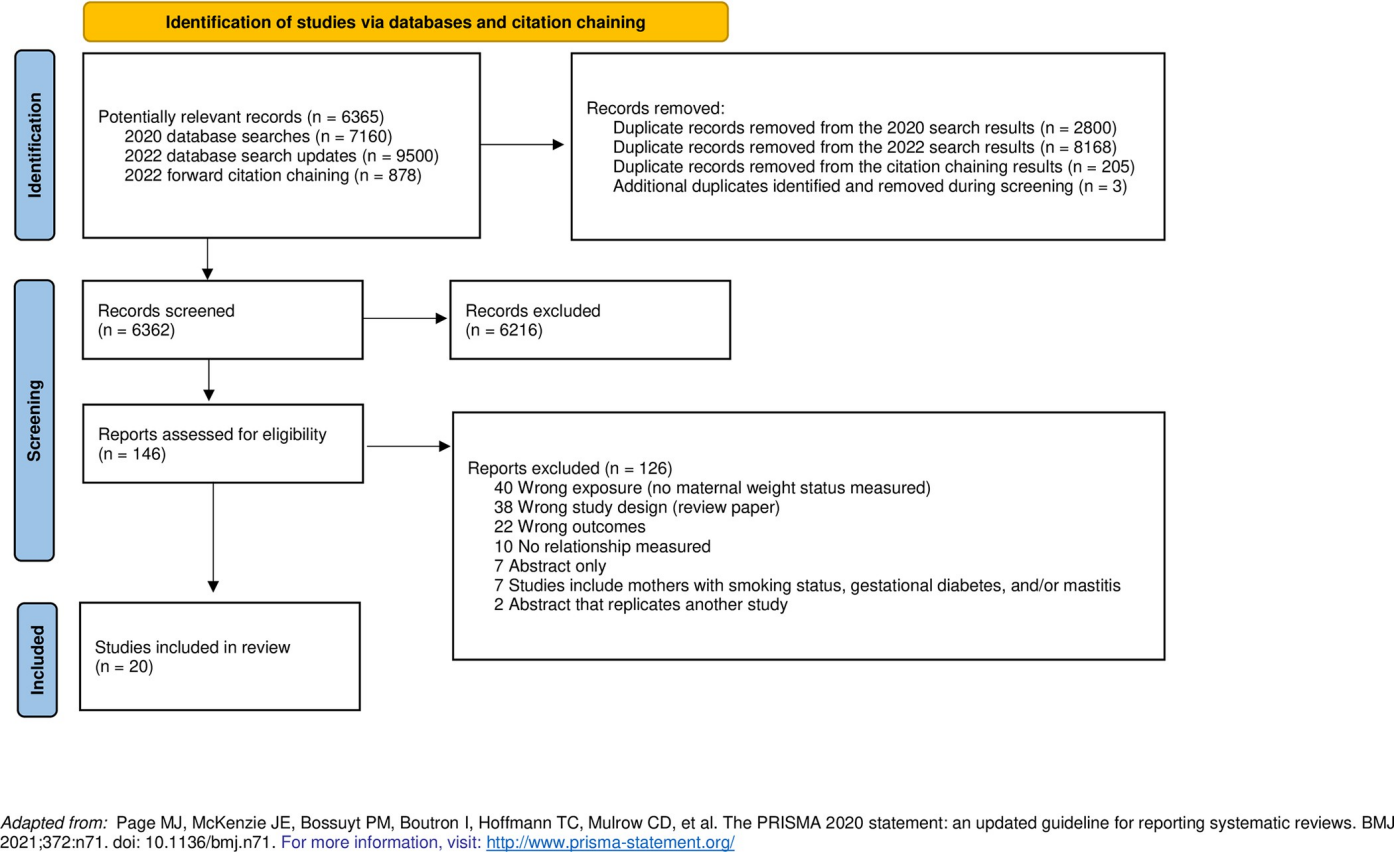
PRISMA 2020 flow diagram for new systematic reviews which included searches of databases and other sources.

Two reviewers (KD & VH) independently screened the results of the database searches in Covidence [28] in two phases: title-abstract screening and full-text screening. Discrepancies were resolved by a third reviewer (UMM). Reviewers contacted the original authors to attempt to gain access to any important, missing information. A PRISMA flow chart was created to record the search, including the results of the entire search, inclusions, and exclusions (**Fig 1;** [29]). Data extraction was done independently by two reviewers (KD & VH) within Covidence, based on a form developed for the purpose by the authors. Disagreements were resolved by a third party, UMM.

We chose not to include a risk of bias assessment due to the exploratory nature of this area of research. We present the results in a table of key findings (**Table 1**) and in a narrative format. For a table with all extracted data, including additional characteristics of each of the studies, see **S3 Table**.

**Table 1:** Key findings of included studies.

First Author and Year	Maternal weight status measure(s)	Study Design, Study Objectives, Location, Sample size	Maternal characteristics	Method of collection, method of microbiome analysis, taxonomic level(s) examined, method of statistical analysis, confounders adjusted for	Key Findings
Cabrera-Rubio et al. 2012 [30]	Prepregnancy weight, prepregnancy BMI, GWG	Longitudinal To identify pre- and post-natal factors that influence bacterial communities in human milk. Finland N = 18	Milk sampled at 2 days, 1 month and 6 months postpartum (Colostrum, mature milk) Maternal age = 32 +/- 5.12 Gestational age = 40.40 +/- 1.1 Prepregnancy weight (kg) = 76.11 50% vaginal deliveries	Manual expression 16S rRNA sequencing (V1-V2 regions), qPCR Genus Principal components analysis; Pearson’s r correlation; mixed models; rarefaction curves No adjustment for confounders	• Women with OB and EGWG had more homogenous milk bacterial composition compared to normal weight women (no effect size or p-value reported). • Maternal BMI was positively associated with *Lactobacillus* in colostrum (r = 0.600, p = 0.026) and *Staphylococcus* at 6 months (r = 0.560, p = 0.038), and negatively associated with *Bifidobacterium* at 6 months (r = -0.651, p = 0.012). • Over the first 6 months, women with OB had higher total bacterial counts (ratio: 0.34 [95% CI: 0.08–0.60]; p = 0.011); higher *Staphylococcus* (ratio: 0.62 [95% CI: 0.30–0.93]; p = 0.0001); higher *Lactobacillus* (ratio: 0.52 [95% CI: 0.02–2.02]; p = 0.038); and lower *Bifidobacterium* (ratio: -0.48 [95% CI: -0.78–0.18]; p = 0.002) compared to normal weight women.Women with EGWG had higher *Staphylococcus* (p = 0.09) and *Staphylococcus aureus* abundances at 1 month (p = 0.03); higher *Lactobacillus* at 6 months (p = 0.03); lower *Bifidobacterium* at 6 months (p = 0.03), compared to women with normal GWG.
Collado et al. 2012 [31]	Prepregnancy weight, prepregnancy BMI, GWG	Longitudinal To assess the relationship between cytokines and milk microbiota, and to explore how maternal factors influence these. Finland N = 56	Milk sampled at 1–2 days, 1 month and 6 months postpartum (Colostrum, mature milk) Maternal age = 30.23 +/- 4.90 Prepregnancy weight (kg) = 72.44 +/- 15.30 Prepregnancy BMI (kg/m^2^) = 21.78 +/- 5.38 76.8% vaginal deliveries	Manual expression qPCR Genus Mann-Whitney U Tests; Chi-Squared tests; mixed models; Spearman’s correlation No adjustment for confounders	• Women with OW had higher *Staphylococcus* and lower *Bifidobacterium* bacteria at 1 month (no effect sizes reported; p = 0.023 and p = 0.009, respectively) and 6 months (no effect size reported; p = 0.023 and p = 0.040, respectively). • Women with OW had higher total bacteria counts (ratio = 0.34, p = 0.011 [95% CI: 0.08–0.060]), higher *Staphylococcus* (ratio = 0.34, p = 0.0001 [95% CI: 0.30–0.93]), higher *Lactobacillus* (ratio: 0.52, p = 0.038 [95% CI: 0.02–2.02]) and lower *Bifidobacterium* (ratio = -0.48, p = 0.002 [95% CI: -0.78 to -0.18]) over the first 6 months. • EGWG was associated with higher *Staphylococcus* in colostrum (no effect size reported; p = 0.050), lower *Bifidobacterium* at 1 month (no effect size reported; p = 0.030), and fewer *Bifidobacterium* bacteria during lactation than women with normal GWG (*b* = -0.42, p = 0.004, [95% CI: -0.71 to -0.14]).
Davé et al., 2016 [32]	Prepregnancy BMI	Cross-sectional To describe the microbiome composition of mother-child dyads and explore its relationship with maternal and childhood obesity. United States N = 10	Milk sampled at 2–4 days postpartum (colostrum) Maternal age = 25.4 +/- 3.4 100% vaginal deliveries	Breast pump 16S rRNA sequencing (V4 region) Genus Pearson’s correlation, Principal components analysis No adjustment for confounders	• Prepregnancy BMI was negatively associated with *Streptococcus* abundance (r = -0.67, p = 0.048). • Prepregnancy BMI was positively associated with microbial diversity (r = 0.77, p = 0.016).
Li et al., 2017 [33]	Postpartum BMI	Cross-sectional To characterize the milk microbiome of East Asian women and to assess whether delivery mode impacts the milk microbiota. Taiwan and mainland China, N = 133 (Taiwan = 31, China = 102)	Milk sampled at random time points for each participant (Mean milk sampling month: 6.1 +/- 4.0) (colostrum, transitional milk, mature milk) Maternal age = 28.5 years +/-4.6 Three BMI (kg/m^2^) groups: <18.5 (n = 12), 18.5–25.0 (n = 87), and >25.0 (n = 32) 39% vaginal deliveries	Breast pump 16S rRNA pyrosequencing, (V1-V2 region) Family, Genus No adjustment for confounders	• No significant differences in the abundances of predominant bacterial families among three different (postpartum) maternal BMI groups (no effect size or p-value reported).
Williams et al., 2017 [34]	Prepregnancy BMI, Postpartum BMI	Longitudinal To characterize the human milk microbiome and describe associations with maternal diet, time postpartum, delivery mode and maternal BMI. United States N = 21	Milk sampled at 2, 5, and 10 days, and 1, 2, 3, 4, 5, and 6 months postpartum (Colostrum, transitional milk, mature milk) Maternal age = 30 +/- 4 Prepregnancy weight (kg) = 64 +/- 7	Breast pump 16S rRNA sequencing, (V1-V3 region) Phylum, Family, Genus Generalized linear mixed models; Spearman rank-order correlation analysis No adjustment for confounders	• No association between categorical prepregnancy BMI on the relative abundance of the predominant bacterial phyla (no effect size or p-value reported). • Women with OW and OB had a higher abundance of *Granulicatella* in milk than normal weight women (1.8% +/- 0.6% compared with 0.4% +/- 0.2%, respectively; p<0.05). • Current (postpartum) BMI was negatively correlated with *Bacteroides* (r = -0.46, p = 0.037).
Asbury et al., 2018 [35]	Prepregnancy BMI	Longitudinal To characterize the milk microbiome composition from mothers of preterm infants (born <1250 g) over the first 8 weeks (2 months) postpartum. Canada N = 30	Milk sampled weekly over first 8 weeks postpartum (colostrum, transitional milk, mature milk)	16S rRNA sequencing (V4 region) Genus Linear and Poisson regressions Adjustment for delivery mode, antibiotic use	• No association between richness and prepregnancy BMI over the first 8 weeks postpartum (no effect size or p-value reported). • Women with normal weight had greater microbial evenness (Shannon diversity) over the first 8 weeks compared to women with OW and OB (0.13 vs -0.07 per week, p = 0.0002).
Li et al., 2017 [36]	Postpartum BMI	Cross-sectional To explore how maternal and infant characteristics influence milk bacterial composition. Guatemala N = 76	Milk sampled at 5–46 days and 4–6 months postpartum (colostrum, transitional milk, mature milk)	16S rRNA sequencing (region not specified) Phylum, Family No adjustment for confounders	• Women with normal BMI had higher *Alphaproteobacteria* and *Betaproteobacteria* compared to women with OW and OB (no effect size or p-value reported).
Boix-Amoros et al., 2019 [37]	Prepregnancy BMI	Cross-sectional To determine whether milk mycobiota (fungal communities) is influenced by geographic location and maternal characteristics, and how the mycobiome is related to milk bacterial composition. Spain, Finland, South Africa and China N = 80 (20 per country)	Milk sampled at 1 month postpartum (mature milk) Maternal age = 33.52 +/-4.87 Prepregnancy BMI (kg/m^2^) = 24.06 +/- 3.85 50% vaginal deliveries	Manual expression 18S rRNA sequencing rRNA sequencing (region not specified), 5.8S rRNA sequencing rRNA sequencing (ITS1 region), qPCR, fungal culturing Phylum, Genus Multivariate analysis with linear model Adjusted for maternal age, pre-delivery maternal BMI, and antibiotic use at delivery	• Prepregnancy BMI was not associated with overall human milk bacteriome or mycobiome composition (no effect size or p-value reported). • Prepregnancy BMI was positively associated with *Davidella* and *Sistotrema* abundance among South African women; positively associated with *Staphylococcus* and *Bacilli* abundance among Spanish women; negatively associated with *Ascomycota* and *Sistotrema* among Chinese women; negatively associated with unclassified *Bacilli* in Finnish women (no effect size reported; p<0.05).
Ding et al., 2019 [38]	Prepregnancy BMI	Cross-sectional To a) determine which members of the milk bacteriome are culturable (and thus, viable) on selective media and b) determine the geographic sources of microbes in human milk. China N = 89	Milk sampled at 42 days postpartum (mature milk) Maternal age range = 20–35 years Prepregnancy BMI (kg/m^2^), by region: Northeast China = 20.8; South China = 20.8; Northwest China = no data; East China = 21.5 North China = 20.8	Manual expression, breast pump Culturing; qPCR; 16S rRNA sequencing (V3-V4 regions) Genus, Species One-way ANOVA; Kruskal-Wallis test; Pearson’s correlation No adjustment for confounders	• Prepregnancy BMI was not associated with the relative abundances of the four dominant genera (*Lactobacillus*, *Streptococcus*, *Staphylococcus and Enterococcus* (no effect size or p-value reported). • Postpartum BMI was positively associated with *Staphylococcus* (r = 0.325, p = 0.085), and negatively associated with *Lactobacillus* (r = -0.204, p = 0.85) and *Streptococcus* (r = 0.194, p = 0.103).
Lundgren et al., 2019 [39]	Prepregnancy BMI, GWG, GWG category	Cross-sectional To identify how breastfeeding-associated microbial communities are associated with maternal and infant characteristics. United States N = 155	Milk sampled at 6 weeks (1.5 months) postpartum (mature milk) Maternal age = 32.4	Manual expression 16S rRNA sequencing (V4-V5 regions) Phylum, Family, Genus, Species Multinomial logistic regression; Linear regression; Kruskal-Wallis rank sum test and Dunn’s tests; PERMANOVA Adjusted for postpartum collection week, gestational weight gain, and antibiotic use (before 4 months postpartum)	• Higher prepregnancy BMI was associated with increased odds for harboring a milk microbiome “type” (BMT1) with high *Staphylococcus* and *Streptococcus* (Odds Ratio [OR] = 1.13 [95% CI: 1.02–1.24]), as well as with a type of milk microbiome with high *Acinetobacter* (BMT3) compared to a type with high *Staphylococcus* and high diversity (BMT2) (OR = 1.12 [95% CI: 1.01–1.25]). • Increased GWG (per 10 lbs.) was associated with decreased of having a milk microbiome type with high *Staphylococcus* and *Streptococcus* (BMT1) vs. a type with high *Staphylococcus* and high diversity (BMT2) (OR = 0.66 [95% CI: 0.44–1.00]). • GWG (per 10 lbs.) was positively associated with alpha diversity (Simpson’s diversity; *b =* 0.23, p = 0.022). • Prepregnancy BMI and GWG were associated with milk microbiome cluster membership (no effect size; p = 0.042 and p = 0.050, respectively).
Moossavi et al., 2019 [40]	Prepregnancy BMI	Cross-sectional To determine a) the profile of milk microbiota in a large sample of healthy mothers and b) its association with maternal, early life, and non-microbial aspects of milk composition. Canada N = 393	Milk sampled at 3–4 months postpartum (mature milk) Maternal age: ages 20–30 = 25.3%; ages 30–40 = 68.4%; ages >40 = 6.3% Prepregnancy BMI (kg/m^2^) = 24.3 +/-5.2	Manual expression, breast pump 16S rRNA sequencing (V4 region) Phylum, Order, Family, Genus, Species Multiple linear regression; redundancy analysis; structural equations modeling No adjustment for confounders	• Postpartum BMI was not associated with alpha diversity (Mean normal weight richness = 147 +/- 43; Overweight/obese diversity = 15.6 +/- 8.9). • Postpartum BMI was associated with overall milk composition (explaining <1% of variation). • Postpartum BMI was inversely associated with diversity within Proteobacteria phylum and positively associated with diversity within Firmicutes phylum (no effect size or p-value reported).
Asbury et al., 2020 [41]	Prepregnancy BMI	Longitudinal To examine a) the temporal dynamics of milk microbiota in mothers of preterm infants and b) the relationship between milk microbiota and maternal characteristics Canada N = 86	Milk sampled weekly over first 8 weeks postpartum (mature milk) Mean maternal age = 33.4 +/- 4.8 Prepregnancy BMI (kg/m^2^) = 25.2 +/- 5.5 38% vaginal deliveries	Manual expression, breast pump 16S rRNA sequencing (V4 region) Genus Linear mixed effects models; repeated measures Poisson regression models Adjusted for postpartum week, gestational age, delivery mode, sequencing batch effects and antibiotic use	• While alpha diversity increased over the first 8 weeks postpartum in women with normal BMIs, this increase was delayed in women with OW and OB (no effect size reported; p = 0.04). • In the first 6 weeks, women with OB have greater milk microbial richness than women with OW and normal BMIs (no effect size reported; p = 0.0008). • Women with OB had greater *Staphylococcus* and lower *Acinetobacter*, *Streptococcus* and *Prevotella* compared with women with OW and normal BMIs (no effect size reported; p<0.05). • Women with normal BMI had greater *Corynebacterium* and *Escherichia-Shigella* abundance over time (no effect size reported; p<0.05), and an inverted parabolic shift in *Streptococcus* over the first 6 weeks (no effect size reported; p = 0.02), compared with women with OW and OB.
LeMay-Nedjelski et al., 2020 [42]	Prepregnancy BMI, postpartum BMI	Cross-sectional To investigate the association between maternal characteristics and the milk microbiome. Canada N = 113	Milk sampled at 3 months postpartum (mature milk) Maternal age = 34.2 +/- 4.2 ​​Prepregnancy BMI (kg/m^2^) = 24.3 +/- 4.6 Postpartum BMI (kg/m^2^) = 26.4 +/- 5.2 56.6% vaginal deliveries	Breast pump 16S rRNA sequencing (V4 region) Genus Multivariable linear and Poisson regressions Adjusted for maternal glucose tolerance status, delivery mode, sequencing batch effects	• No association between prepregnancy and postpartum BMI with alpha diversity (Chao diversity: p = 0.859, p = 0.945, respectively; Shannon diversity: p = 0.7143, p = 0.8905, respectively). • Milk microbiome clustered according to pre-pregnancy BMI, even after covariate adjustment (effect size and p-value reported in supplementary information). • Women with OW had greater *Brevundimonas* compared with women with normal (IRR: 9.56 [95% CI: 2.17–42.22]) and overweight BMIs (IRR: 8.89 [95% CI: 2.29–34.57]).
Treven et al., 2019 [43]	Postpartum BMI	Cross-sectional To characterize the human milk microbiota with 16S rRNA sequencing and approaches using cultivation and matrix-assisted laser desorption/ionization mass spectrometry (MALDI-TOF MS) Slovenia N = 32	Milk sampled at 3–8 weeks postpartum (transitional milk, mature milk)	Manual expression, breast pump qPCR; 16S r RNA sequencing (V3-V4 regions); cultivation/matrix-assisted laser desorption/ionization mass spectrometry (MALDI-TOF MS); Sanger sequencing Phylum, Genus Pearson’s correlation; linear discriminant analysis (LefSe) No adjustment for confounders	• Maternal BMI was not significantly associated with the specific patterns in HMM, regardless of use of cultivation approaches or 16S rRNA sequencing (no effect size or p-value reported).
Pace et al., 2021 [44]	Postpartum BMI	Cross-sectional To characterize the associations between milk lactose, oligosaccharides and protein with the milk and infant fecal microbiome across 11 geographic sites. Ethiopia, The Gambia, Peru, Spain, Sweden, United States N = 357	Milk sampled at 64.6 +/-21.9 days (mature milk) Maternal age = 27.4 +/- 6.1 Postpartum BMI (kg/m^2^): 24.2+/-4.6	Breast pump 16S rRNA sequencing (V1-V3 region) Genus Dirichlet multinomial mixtures modelling (to identify clusters of microbiome types, or “lactotypes”); p Kruskal-Wallis test; Wilcoxon rank test, Chi-squared test; Multiple regression with envfit package in R No adjustment for confounders	• Maternal BMI was not associated with the milk microbiome composition (no effect size or p-value reported). • Microbial lactotypes were associated with maternal BMI (p<0.001; FDR p = 0.002).
Bayaga et al., 2021 [45]	Postpartum BMI	Longitudinal To examine how maternal factors influence the total plate count, Staphylococci, Lactobacilli, and Bifidobacteria in milk of lactating women across the first 4 months postpartum. Philippines N = 34	Milk sampled from 0–4 months postpartum (colostrum, transitional milk, mature milk) Maternal age = 25.59 +/- 4.71 Postpartum BMI (kg/m^2^): 32.35% overweight, 47.06% normal weight, 20.59% underweight	Manual expression Culturing Genus Multiple linear regression; Chi-squared test No adjustment for confounders	• Women with overweight BMI had significantly lower counts of Lactobacilli and Bifidobacteria for most months of the study (no effect size reported; p = 0.017).
Yan et al., 2021 [46]	Postpartum BMI	Cross-sectional To examine whether *Bifidobacterium* phylotypes in milk co-occurred in a persistent manner within mother-infant dyads China N = 25	Milk sampled at 7–720 days postpartum (transitional milk, mature milk) Postpartum BMI (kg/m^2^): 64% normal weight; 16% overweight: 12% obese; 4% severe obesity 100% vaginal deliveries	Breast pump 16S rRNA sequencing (V4-V5 region) Phylum, Family Spearman’s correlation; PERMANOVA No adjustment for confounders	• No significant association between family-level microbiome structure and maternal BMI (R^2^< 0.2, p> 0.05).
Butts et al., 2020 [47]	Postpartum BMI	Cross-sectional To examine the milk microbiome, immune modulatory proteins in milk, and the fecal microbiome in mother-infant dyads. New Zealand N = 78	Milk sampled at 6–8 weeks postpartum (mature milk) Maternal age = 31 +/- 5 Postpartum BMI (kg/m^2^) = 27+/-5 82.1% vaginal deliveries	16S rRNA sequencing (V3-V4 region) Phylum, Genus Kruskal-Wallis non-parametric analysis of variance (ANOVA) No adjustment for confounders	• No significant differences in bacterial composition of milk (at genus and phylum level) based on BMI categories (normal, OW and OB; effect size not reported).
Cortes-Macias et al., 2021 [48]	Prepregnancy BMI, GWG	Cross-sectional To characterize the impact of feeding practices and maternal prepregnancy BMI and weight gain on the composition of the milk microbiome. Spain N = 136	Milk sampled within 30 days postpartum (colostrum, transitional milk) Maternal age = 34.44 +/- 3.79 Prepregnancy BMI (kg/m^2^) = 22.84 (range: 21.01–25.39) GWG (kg) = 12 (range: 9.5–15) Median gestational age: 40 62.5% vaginal deliveries	Breast pump qPCR; 16S rRNA sequencing (V3-V4 region) Genus Spearman’s correlation; PERMANOVA; discriminant of Principal Components Analysis (DAPC); Redundancy analysis (RDA); t-test; Mann-Whitney U test; Multivariable Poisson regression models Adjusted for: birth mode, feeding practices at 1 month (Poisson regression models); diversity and richness values were adjusted for total bacterial load	• Women with normal BMI had higher prevalence of *Bifidobacterium* (incidence rate ratio: 4.67 (2.53–8.64)), *Ralstonia* (1.16 (1.03–1.32)), but lower incidence of *Staphylococcus* (0.89 (0.83–0.96)) than women with OW BMI; analyses adjusted for mode of birth and feeding practices at 1 month. • Women with OW BMI had higher total bacterial counts (log10 bacterial gene copies/mL of milk) than women with normal BMI (normal BMI: 6.70 (5.77–7.17) vs. OW BMI: 6.94 (6.42–7.44); p = 0.031). • Women with higher prepregnancy BMI had lower Shannon diversity and richness (diversity: rho = -0.05, p = 0.582; richness: rho = -0.03, p = 0.753). • Prepregnancy BMI was associated with overall milk microbiome composition in exclusively breastfeeding women, but not in women who were mixed-feeding (EBF: Adonis Bray-Curtis R^2^ = 0.0254, p = 0.05; MF; Adonis Bray-Curtis R^2^ = 0.022, p = 0.029); however, the association between prepregnancy BMI and overall composition was not observed in women with OW BMI (RDA test variance = 1.56, p = 0.928; Adonis Bray-Curtis R^2^ = 0.0183, p = 0.78). • Women with normal GWG had lower diversity (p = 0.026), greater incidence of *Bifidobacterium* (incidence rate ratio: 3.20 (1.71–5.98)), *Streptococcus* (1.38 [95% CI: 1.27–1.51]), and lower *Ralstonia* (0.53 [95% CI: 0.46–0.61]) compared to women with EGWG. • Women with normal GWG had significant differences in the milk microbiome community according to feeding practices (RDA test variance = 1.3, p = 0.014; Adonis Bray-Curtis R^2^ = 0.015, p = 0.111); however, this was not observed in women with EGWG (RDA test variance = 1.43, p = 0.087; Adonis Bray-Curtis R^2^ = 0.0189, p = 0.109). • Mixed-feeding women with normal GWG had marginally higher abundances of *Staphylococcus* (p = 0.049) and lower *Pseudomonas* (p = 0.019) than other women. • Exclusively breastfeeding women with normal BMI had higher diversity and richness (this was also observed in mixed-feeding, normal BMI women; no effect size or p-value reported), higher relative abundance of *Bifidobacterium* (p = 0.033) and lower *Pseudomonas* (p<0.01) compared to other groups.
Sanjulian et al., 2021 [49]	Postpartum BMI, GWG	Cross-sectional To characterize the milk microbiome and examine the impact of lactation time on milk microbiome diversity in healthy Spanish women Spain N = 99	Milk sampled at 2 weeks to 5 years (transitional milk, mature milk) Maternal age = 35.46 +/- 4.02 Postpartum BMI (kg/m^2^) = 24.48 +/- 3.85 GWG (kg) = 13.25 +/- 3.63 Gestational age = 39.76 +/-1.33 86.21% vaginal deliveries	Breast pump qPCR; 16S rRNA sequencing (V2, V3, V4, V6–7, V8, and V9 regions) Phylum, Genus Pearson’s correlation No adjustment for confounders	• Positive correlation between maternal BMI and *Lactobacillus* (r = 0.277, p = 0.034) and *Enterococcus* (r = 0.325, p = 0.046).

Abbreviations: GWG = Gestational weight gain; EGWG = Excess gestational weight G = gain; OW = Overweight; OB = Obese, qPCR = quantitative polymerase chain reaction, PERMANOVA = permutational analysis of variance; ASV = Amplicon sequence variant, IRR = incidence rate ratio, OR = odds ratio. Gestational age is in weeks, maternal age is in years, BMI is in kg/m^2^, and GWG/EGWG is in kg, unless otherwise noted. All studies followed Institute of Medicine guidelines [7] to categorize GWG (e.g., excessive vs. normal GWG). All values are mean +/- standard deviation, unless otherwise indicated. Information is presented only if it was reported in the original study.

## Results

Results of key findings from extracted data are presented in **Table 1**. The composition of the milk microbiome composition, as observed by each study, is presented in **Table 2**.

**Table 2:** Composition of the milk microbiome by study.

First Author and Year	Composition of milk microbiome
Cabrera-Rubio et al., 2012 [30]	Genus (colostrum): *Weisella*, *Leuconostoc*, *Staphylococcus*, *Streptococcus*, *Lactococcus* Genus (1–6 months postpartum): Lactic acid bacteria were the most abundant in 1- and 6-month samples; bacteria typically found in the oral cavity *(Veillonella*, *Leptotrichia and Prevotella*) increased over time.
Collado et al. 2012 [31]	Genus: *Bifidobacterium*, *Staphylococcus*, *S*. *aureus*, *Lactobacillus*, *Enterococcus*, *Streptococcus* Species: *Akkermansia muciniphila*, *Clostridium coocoides group*
Davé et al., 2016 [32]	Genus: *Streptococcus* (73.8%), *Staphylococcus* (10.9%).
Li et al., 2017 [33]	Family: Streptoccocaceae (24.4%), Pseudomonadaceae (14.0%), Staphylococcaceae (12.2%), Lactobacillaceae (6.2%) and Oxalobacteraceae (4.8%)
Williams et al., 2017 [34]	Phylum: Firmicutes (85.1% +/- 1.2%), Actinobacteria (5.9% +/- 0.9%), Proteobacteria (2.3% +/- 0.3%), and Bacteroidetes (1.3% +/- 0.3%) Genus: *Streptococcus* (45.2% +/- 2.6%) and *Staphylococcus* (25.3% +/- 2.6%)
Asbury et al., 2018 [35]	Genus: *Staphylococcus* (50.1% +/- 27.1%), *Pseudomonas* (17.6% +/- 14.8%), *Acinetobacter* (10.6% +/- 19.1%), *Corynebacterium* (4.7% +/- 7.0%), *Streptococcus* (2.9% +/- 9.7%)
Li et al., 2017 [36]	No information
Boix-Amoros et al., 2019 [37]	Phylum: Basidiomycota (58.65%) and Ascomycota (41.03%) Genus: *Malassezia* (40.6%), *Davidella* (9.0%)
Ding et al., 2019 [38]	Milk microbiota clustered into three groups, including groups enriched with *Enterococcus* (Group 1), *Streptococcus* (Group 2) and *Staphylococcus* (Group 3).
Lundgren et al., 2019 [39]	Genus: *Acinetobacter* (14.3%), *Streptococcus* (13.7%), *Pseudomonas* (11.3%), *Staphylococcus* (11.0%), Enterobacteriaceae (8.23%), *Bacteroides* (1.07%), *Bifidobacterium* (0.651%) Milk microbiome clustered into four “breastfeeding microbiome” types (BMTs). BMT1 was characterized by high *Streptococcus* and *Staphylococcus*, BMT2 had high *Streptococcus* and high alpha diversity, BMT3 had high *Acinetobacter*, as well as high median abundance of Enterobacteriaceae and Pseudomonas, and BMT4 has low alpha diversity and high *Acinetobacter*.
Moossavi et al., 2019 [40]	Phylum: Proteobacteria (67% +/- 24%), Firmicutes (26% +/- 22%), Actinobacteria (4% +/- 4%) and Bacteroidetes (1% +/- 3%) Genus: *Streptococcus* (16% +/- 17%), *Ralstonia* (5% +/- 3%), and *Staphylococcus* (5% +/- 11%).
Asbury et al., 2020 [41]	Genus: *Staphylococcus*, *Acinetobacter*, *Pseudomonas*, *Corynebacterium*, *Streptococcus*, *Stenotrophomonas*, *Prevotella*, *Escherichia*, *Shigella*, *Finegoldia*, and *Lactobacillus*
LeMay-Nedjelski et al., 2020 [42]	Phylum: Proteobacteria (58.6% +/- 27.3%), Firmicutes (35.6% +/- 26.3%), Actinobacteria (4.1% +/- 4.7%), Bacteroidetes (1.4% +/- 2.7%), Fusobacteria (0.1% +/- 0.3%) Genus: *Pseudomonas* (43.4% +/- 26%), *Streptococcus* (30.6% +/- 25.3%).
Treven et al., 2019 [43]	Phylum: Firmicutes, Proteobacteria, Actinobacteria *Genus*: *Staphylococcus* (36.0%), *Streptococcus* (35.6%), *Acinetobacter* (8.3%), *Gemella* (2.5%), *Corynebacterium* (1.3%), *Veillonella* (0.09%) Diversity: Observed species (OTUs): 33.26 (range 10–70); mean Chao diversity: 38.37 +/- 12.66
Pace et al., 2021 [44]	Genus: *Staphylococcus* (28%), *Streptococcus* (26%), *Corynebacterium* (6%), *Propionibacterium* (5%), unclassified genera from the family Xanthomonadaceae (3%), and *Lactobacillus* (3%). Milk microbiome clustered into four lactotypes—L1, L2, L3 and L4. L1 was mainly individuals from Americas and Europe, while L2 and L3 were largely from Africa. L4 was rural Ethiopia. All lactotypes were dominated by *Streptococcus*, *Propionibacterium*, *Lactobacillus* and *Corynebacterium*. *Bifidobacterium* was an indicator taxon for L4. Diversity: Highest Shannon diversity and number of observed ASVs in L4 and lowest in L3.
Bayaga et al., 2021 [45]	Genus: Staphylococci (3.17–4.37 log CFU/ml), Lactobacilli (2.74–3.76 log CFU/ml), Bifidobacteria (2.98–4.30 log CFU/ml) Other: Total plate count (TPC) (3.94–5.22 log CFU/ml), total coliform counts: <1.00–3.12 log CFU/ml
Yan et al., 2021 [46]	Phylum: Proteobacteria (46.5%) Family: Enterobacteriaceae (25.5%); Streptococcaceae (19.3%); Pseudomoadacaea (2.2%) Diversity: Observed ASVs: 204 +/- 109 species
Butts et al., 2020 [47]	Genus: *Bifidobacterium* (values not reported) Family: *Ruminococcacceae*, *Lacnospiraceae* (values not reported)
Cortes-Macias et al., 2021 [48]	Phylum: Firmicutes (66.3%) and Proteobacteria (28.8%), Actinobacteria (3.7%), Bacteroidetes (1.18%) Genus: *Streptococcus* (29.2%), *Staphylococcus* (27.8%), *Ralstonia* (10.1%), *Acinetobacter* (9.6%)
Sanjulian et al., 2021 [49]	Genus: *Streptococcus*: 4.10 +/- 0.80 logCFU/mL; *Prevotella*: 3.78 +/- 0.93 logCFU/mL; *Bacteroides*: 3.43 +/- 1.02 logCFU/mL; *Lactobacillus*: 3.10 +/- 0.56 logCFU/mL; *Enterococcus*: 2.67 +/- 0.66 logCFU/mL; *Staphylococcus*: 2.61 +/- 0.46 logCFU/mL Phylum: Firmicutes (4.24 +/- 0.87 logCFU/mL), Bacteroidetes (3.80 +/- 0.88 log CFU/mL), Actinobacteria (3.42 +/- 0.78 log CFU/mL), Proteobacteria (3.17 +/-1.15 logCFU/mL) Diversity: Milk bacterial diversity increased from 1.3–41.6 months (no effect size or p-value reported)

Abbreviations: CFU = colony-forming unit. Percentages represent mean relative abundance of taxa +/- standard deviation

## Study characteristics

We found twenty studies (**Tables 1 and 2; S3 Table**) investigating associations between the human milk microbiome and maternal weight status. Only 4 of the 20 studies explicitly aimed to investigate relationships between maternal weight status and the milk microbiome [32, 34, 42, 48]. The remaining 16 out of 20 studies investigated weight status-milk microbiome relationships as part of their exploration of the data, not as a specific objective. Maternal weight status was commonly measured in three ways: prepregnancy BMI, postpartum BMI, and GWG. Most studies (17/20) characterized the milk microbiome through 16S rRNA sequencing, while some (6/20) measured specific microbial abundances through qPCR and others through culturing of microbes on selective media, which was often combined with other sequencing techniques (Table 1). One study [37] utilized 18.5S and 5.8S sequencing, which are techniques specific for the assessment of fungal communities. Two studies [37, 38] characterized milk microbial communities through the cultivation of bacteria and fungi on selective media.

In the extracted studies, the time of milk collection ranged from 1–2 days to 5 years postpartum. Human milk composition changes over the lactation period and accordingly, lactation is divided into three stages: colostrum (1–5 days), transitional milk (5–21 days), and mature milk (21+ days; [50]). Just under half of included studies only collected mature milk (8/20), while other studies sampled both colostrum and mature milk (2/20), colostrum only (1/20), colostrum and transitional milk (1/20), transitional and mature milk (3/20), and colostrum, transitional, and mature milk together (5/20). Most studies utilized a cross-sectional study design (14/20), while others (6/20) examined the milk microbiome longitudinally over the course of lactation. Six (6/20) studies collected milk via manual expression, seven (7/20) studies collected milk with an electric or manual breast pump, two studies (2/20) included both manual and breast pump expression, and one study did not report how milk was collected. Nearly all included studies (14/20) had participants who took antibiotics in the gestational and/or postpartum period, three studies (3/20) excluded women who took antibiotics from analyses or from participating, and three (3/20) other studies did not report any information about antibiotic use. Studies had sample sizes ranging from 10 to 393 lactating women, and were conducted in various global locations, including the U.S, Canada, Finland, China, Taiwan, Guatemala, Spain, New Zealand, the Philippines, and South Africa, to name a few.

The studies extracted in this review differed in how they adjusted for confounding variables in their statistical analyses of associations between maternal weight status and milk microbiome composition. While 14 studies did not report any adjustment for confounders, 6 studies did report adjustment. These confounders adjusted for included postpartum milk collection week, maternal age, antibiotic use, mode of delivery, gestational age, sequencing batch effects, and GWG, among others.

Across all studies, the milk microbiome was composed of similar common bacterial taxonomic groups. Common phyla included Proteobacteria, Firmicutes, Actinobacteria and Bacteroidetes [34, 40, 51]. Common genera included *Staphylococcus* (predominant in 13/20 studies, range of relative abundance: 5–50%), *Streptococcus* (12/20 studies, 2.9–45.2%), *Acinetobacter* (6/20, 3.5–14.3%), *Pseudomonas* (4/20, 11.3–43.4%), *Corynebacterium* (5/20, 1.3–6.0%), and to a lesser extent, *Bifidobacterium* (4/20 studies, <1% abundance). It is important to note that the detection of *Bifidobacterium* is difficult through rRNA sequencing and often depends on the primers used and the type of hypervariable regions of 16S rRNA gene that were sequenced [52]. Only a few studies (6/20) employed specific approaches to detect *Bifidobacterium* and other low-abundance genera [30, 31, 45, 46, 48, 49].

### Maternal weight status and the overall composition and diversity of the milk microbiome

Many included studies (12/20) found that BMI in the prepregnancy and postpartum periods were associated with the milk microbiome, although inconsistently and with low effect sizes. In one of the largest investigations of the milk microbiome to date (n = 393), Moossavi and colleagues [40] found that prepregnancy BMI was associated with the overall composition of the milk microbiome, although explaining <1% of its variation. Among Slovenian women, Treven et al. [43] reported that maternal postpartum BMI was not significantly associated with the specific patterns of the human milk microbiome (no effect size or p-value reported). Pace et al. [44] observed that in a cross-geographic comparison, there was not a significant relationship between postpartum BMI and overall milk microbiome composition (no effect size or p-value reported). Yan et al. [46] did not observe a statistically significant association between family-level milk microbiome community structure and maternal postpartum BMI (R^2^<0.2; p>0.05). Similarly, Williams and colleagues [34] observed no differences in overall composition at the phylum level between women of different prepregnancy BMIs (no effect size reported).

Prepregnancy and postpartum BMI appeared to be negatively associated with the alpha diversity of the milk microbiome, although some studies reported no association. According to Cortes-Macias et al. [48], prepregnancy BMI was negatively associated with alpha diversity (Shannon diversity: rho = -0.05, p = 0.582; richness: rho = -0.03, p = 0.753). Similarly, Cabrera-Rubio et al. [30] reported a negative association: women with obese prepregnancy BMI had more homogenous (less diverse) microbiota than other women (no effect size or p-value reported). Another study by Asbury and colleagues (conference abstract; [35]) demonstrated that over the first 8 weeks postpartum, women with normal prepregnancy BMIs had greater species evenness (i.e., the distribution of the abundances of various taxa [53]) compared to women with overweight or obese prepregnancy BMIs; however, microbial richness, or the number of different species in a sample, was not significantly associated with BMI. In a later study by the same research group, Asbury et al. [41] reported that while milk alpha diversity increased over the first 8 weeks among women with normal prepregnancy BMIs, this increase in diversity was delayed among women with overweight and obese BMIs (p = 0.04, no effect size reported). Finally, one study [42] found no significant differences in richness or diversity by prepregnancy BMI or by 3-month postpartum BMI.

### Maternal weight status and specific taxa in the milk microbiome

Across the studies extracted by our review, maternal weight status appeared to be variably associated with the abundance of specific microbial taxa, particularly with *Staphylococcus*, *Streptococcus*, and *Bifidobacterium*. Higher prepregnancy BMI was associated with greater abundance of the phylum Firmicutes, greater abundance of *Staphylococcus*, lower abundance of *Bifidobacterium*, and lower *Streptococcus* (although this latter relationship was more variable). At the genus level, Davé and colleagues [32] observed in a small, cross-sectional sample of Mexican-American women that prepregnancy BMI was negatively associated with *Streptococcus* abundance (r = -0.67; p = 0.048) and positively associated with alpha diversity (r = 0.77; p = 0.016). Similarly, Cortes-Macias and colleagues [48] observed that compared to women with overweight prepregnancy BMI, women with normal BMI had greater *Bifidobacterium* (incidence rate ratio [IRR]: 4.67 [95% CI: 2.53–8.64]), lower *Staphylococcus* (IRR: 0.89 [95% CI: 0.83–0.96]), and greater *Ralstonia* (IRR: 1.16 [95% CI: 1.03–1.32]). Lundgren and colleagues [39] observed that milk microbial composition clustered into four breastfeeding microbiome types (BMTs). Prepregnancy BMI was associated with “breastfeeding microbiome type” (BMT), such that for every one-unit increase in prepregnancy BMI, there was an increased odds for belonging to BMT1 (high *Staphylococcus* and *Streptococcus*, low alpha diversity) versus BMT 2 (high *Streptococcus*, high alpha diversity; [OR = 1.13 (95% CI: 1.02, 1.24)]), and BMT 3 (high *Acinetobacter*) compared to BMT2 (high *Acinobacter* and *Pseudomonas*, low alpha diversity; [OR = 1.12 [95% CI: 1.01–1.25]). That prepregnancy BMI was associated with BMT1 is of interest: BMT1 had a higher abundance of Firmicutes, a phylum that is typically higher in the overweight and obese gut microbiome [54]. Moossavi et al. [40] note that higher prepregnancy BMI was associated with less diversity within the phylum Proteobacteria and greater diversity within the phylum Firmicutes. LeMay-Nedjelski et al. [42] reported that women with obese prepregnancy BMI had lower Proteobacteria (IRR: 0.62 [95% CI: 0.43–0.90]), as well as greater Bacteroidetes (IRR: 3.70 [95% CI: 1.61–8.48]) and Actinobacteria (IRR: 2.34 [95% CI: 1.38–3.98]) compared to women with overweight and normal BMIs.

In terms of postpartum BMI, Ding et al. [38] found that postpartum BMI was positively associated with *Staphylococcus* (Pearson’s r = 0.325) and *Streptococcus* (r = 0.194) and negatively correlated with *Lactobacillus* (r = - 0.204) although these relationships were not statistically significant. Interestingly, the finding that postpartum BMI was positively associated with *Streptococcus* contrasts with the findings of Davé et al. [32], who reported a negative association. Among women in the Philippines, Bayaga et al. [45] reported that women with overweight postpartum BMI had lower counts of *Bifidobacterium* and *Lactobacillus* for the first 4 months postpartum than others (no effect size reported; p = 0.017). LeMay-Nedjelski et al. [42] showed that women with obese BMIs at 3 months postpartum had greater *Staphylococcus* ((IRR: 2.50 [95% CI: 1.09–5.72]) compared to overweight women, greater *Corynebacterium* compared to overweight and normal weight women (IRR: 5.13 [95% CI: 1.79–14.70], and greater Actinobacteria (IRR: 2.34 [95% CI: 1.38–3.98]) compared to overweight and normal weight women. Focusing on class-level differences, Li et al. [36] observed that among Mayan women in Guatemala, a normal postpartum BMI was associated with higher proportions of the classes *Alphaproteobacteria* and *Betaproteobacteria* (no effect size or p-value reported).

These relationships between BMI and *Staphylococcus*, *Streptococcus*, and *Bifidobacterium* also appear to hold over the course of lactation. Collado and colleagues [31] found that compared to women with normal BMI, women with overweight BMI harbored higher counts of *Staphylococcus*-group bacteria at 1 month (median, overweight/obese women (BMI > 25/kg/m^2^) = 4.94 gene copies/mL milk; median, “normal”/underweight women (BMI < 25 kg/m^2^) = 4.40 gene copies/mL milk). The study also found that women with overweight/obese BMI had marginally lower counts of *Bifidobacterium*-group bacteria at both 1 month (median, overweight/obese women = 5.30; median, “normal”/underweight women = 5.84 gene copies/mL of milk) and 6 months (median, overweight/ obese women = 5.19 gene copies/mL; median, “normal”/underweight women = 5.86; [28]). In a similar study, Cabrera-Rubio and colleagues [30] observed that maternal prepregnancy BMI was positively associated with *Lactobacillus* in colostrum (r = 0.6, p = 0.026), positively associated with *Staphylococcus* (r = 0.560, p = 0.038) and negatively associated with *Bifidobacterium* at 6 months (r = 0.651, p = 0.012). This study also found that over the first 6 months postpartum, women with obesity had higher total bacterial counts in milk (ratio: 0.34 [95% CI: 0.08–0.60]; p = 0.011), 0.48 times fewer *Bifidobacterium* (ratio: -0.48 [95% CI: -0.78–0.18]; p = 0.002), 0.62 times more *Staphylococcus* abundance (ratio: 0.62 [95% CI: 0.30–0.93]) and 0.52 times more *Lactobacillus* (ratio: 0.52 [95% CI: 0.02–2.02]; p = 0.038) compared to women with normal prepregnancy BMIs [30]. Asbury et al. [41] reported that women with normal prepregnancy BMIs had increased *Corynebacteria* and *Escherichia*-*Shigella* abundance over time (no effect size reported; p <0.05) and an inverted parabolic shift in *Streptococcus* in the first 6 weeks postpartum, patterns which contrasted with women with overweight and obese BMIs. The same study also found that women with obese prepregnancy BMI had higher *Staphylococcus* and lower abundance of *Acinetobacter*, *Streptococcus* and *Prevotella* in milk compared to women with normal prepregnancy BMIs over the first 6 weeks postpartum (p < 0.05; no effect size reported). Finally, Williams and colleagues [34] found that postpartum BMI was negatively correlated with the genus *Bacteroides* (Pearson’s r = -0.46; p = 0.037).

### Interactions between weight status, feeding mode, and the milk microbiome

One study [48] investigated the possibility that relationships between maternal weight status and the milk microbiome are dependent on feeding mode. Cortes-Macias et al. [48] reported that prepregnancy BMI was associated with the overall milk microbiome composition in exclusively breastfeeding (EBF) women, but not in mixed-feeding (MF) women (EBF: Adonis Bray-Curtis R^2^ = 0.0254, p = 0.05; MF; Adonis Bray-Curtis R^2^ = 0.022, p = 0.029). Notably, this was only observed in women with normal prepregnancy BMI, and not in women with overweight prepregnancy BMI. EBF women with normal prepregnancy BMI also had higher diversity and richness, higher *Bifidobacterium* (no effect size reported; p = 0.033), and lower *Pseudomonas* (no effect size reported; p<0.01) compared to other groups [48]. These findings suggest that relationships between prepregnancy weight status and the milk microbiome may be partly dependent on feeding mode, particularly among women with normal prepregnancy BMIs. Lastly, Butts et al. [47] observed no significant differences in the milk microbiome composition at the genus and phylum level according to postpartum BMI categories (no effect size or p-value reported).

### Gestational weight gain and the milk microbiome

Six studies (6/20) reported relationships between GWG and the milk microbiome. In terms of milk alpha diversity, Cortes-Macias et al. [48] found that GWG was positively associated with alpha diversity; specifically, women with normal GWG had lower milk microbiome diversity (no effect size reported; p = 0.026). Similarly, according to Lundgren et al. [39], for every 10 pounds gained during gestation, the milk microbiome exhibited a 1-unit increase in alpha diversity (Simpson’s diversity, β = 0.23, p = 0.022).

In terms of specific taxa in milk, the results with GWG corroborated other findings involving prepregnancy and postpartum BMI; greater GWG appeared to be linked to greater abundance of *Staphylococcus*, *Streptococcus*, and lower *Bifidobacterium*. For example, Lundgren et al. [39] reported that women with differing GWG (normal vs. excessive) had distinct patterns of milk microbiome composition; increasing maternal GWG (per 10 pounds) was associated with decreased probability of belonging to BMT1 (high *Staphylococcus* and *Streptococcus*, low alpha diversity) vs BMT 2 (high *Streptococcus*, high alpha diversity) (OR = 0.66 [95% CI: 0.44–1.00]). Similarly, Cabrera-Rubio et al. [30] found that women with excessive GWG had more homogenous milk composition and higher counts of *Staphylococcus aureus* (median bacterial count, excessive GWG = 3.79; median bacterial count, normal GWG = 3.00; p = 0.03) at 1 month, higher *Lactobacillus* (median bacterial count, excessive GWG = 6.50; median bacterial count, normal GWG = 5.91; p 0.03) and lower *Bifidobacterium* at 6 months (median bacterial count, excessive GWG = 4.82; median bacterial count, normal GWG = 5.85; p = 0.02). Collado et al. [31] found that excessive GWG was associated with higher *Staphylococcus* in colostrum (p = 0.05; marginally statistically significant) and lower *Bifidobacterium* at 1 month (p = 0.03). Additionally, these authors ran mixed-models and showed that excessive GWG was associated with 0.42 times fewer *Bifidobacterium*-group bacteria in milk throughout the course of lactation (β *=* -0.42, p = 0.004, [95% CI: -0.71 to -0.14]). Cortes-Macias et al. [48] found that compared to women who had excessive GWG, women with normal GWG had greater incidence of *Bifidobacterium* (IRR: 3.20 (1.71–5.98)) and lower *Ralstonia* (IRR: 0.53 [95% CI: 0.46–0.61]); contrasting to findings from other studies, women with normal GWG had greater incidence of *Streptococcus* (IRR: 1.38 [95% CI: 1.27–1.51]). Cortes-Macias and colleagues [48] also found that mixed-feeding women with normal GWG had marginally greater abundance of *Staphylococcus* (p = 0.049) and *Pseudomonas* (p = 0.019).

### Maternal weight status and non-bacterial components of the microbiome

Lastly, while most of the extracted studies (19/20) investigated only the composition of the milk bacteriome, one study described the composition of the milk fungal composition, or *mycobiome*, alongside the bacteriome of milk [37]. This cross-sectional, cross-geographic study (Spain, Finland, South Africa and China) found no associations with overall milk mycobiome composition and prepregnancy BMI. However, prepregnancy BMI was positively associated with the abundance of fungal genera *Davidella* and *Sistotrema* among South African women, and *Staphylococcus* and *Bacilli* abundance in Spanish women, and was negatively associated with *Ascomycota* and *Sistotrema* in Chinese women, and with unclassified *Bacilli* in Finnish women [37].

## Discussion

In this review, we investigated the scope of current knowledge on the relationship between maternal weight status and the composition of the milk microbiome. We conducted a comprehensive search in electronic databases and found 20 studies, 11 of which only reported significant associations, 4 reported both significant and null associations, and 5 reported only null associations between maternal weight status and the milk microbiome. We found that the aims and objectives of these studies varied—while a few studies explicitly focused on delineating maternal weight-milk microbiome relationships, most others reported these associations in their exploration of the data. As in previous reviews [19, 22], milk microbiota was typically characterized by high relative abundances of *Staphylococcus*, *Streptococcus*, *Acinobacter*, and other microbial species that overlap with the communities of the skin microbiome (**Tables 1 and 2).** In general, women who had an overweight or obese BMI in the prepregnancy or postpartum periods, or who experienced excessive GWG, all harbored milk microbiota with higher *Staphylococcus*, higher *Streptococcus* (although this was more variable), lower *Bifidobacterium* abundance, and lower alpha diversity than women with lower BMIs or normal GWG (**Tables 1 and 2**). However, despite these findings, weight status does not appear to be a major predictor of overall milk microbiome composition. In fact, one study reported that maternal BMI explained less than 1% of the variation in the milk microbiome [40], and several others reported that there were no significant associations between maternal weight status and overarching milk microbial composition and community structure [43, 44, 46]. In all, the composition of the milk microbiome may be mildly affected by maternal weight status. We suggest that a) the composition of the maternal gut microbiome, b) maternal diet, and c) breastfeeding and delivery practices may explain this minor effect of weight status on the milk microbiome.

First, the weight-based differences in the milk microbiome reported by the included studies may be explained by differences in the maternal gut microbiome composition attributable to the metabolic effects of overweight or obesity. The composition of the human gut microbiome has been shown to be interlinked with metabolic status; individuals with obesity harbor gut microbiota with decreased abundance of the phylum Bacteroidetes, increased Firmicutes, lower alpha diversity, and altered microbial gene expression favoring increased energy uptake [3, 55–57]. The gut microbiota of women during pregnancy and the postpartum period is associated with maternal weight status. Pregnant women with obesity and excessive GWG harbor higher *Staphylococcus*, including the pathobiont *Staphylococcus aureus*, and lower *Bifidobacterium* abundances in the gut microbiome [6]. Over the course of pregnancy, the maternal gut microbiome appears to shift to a pro-inflammatory, insulin-resistant state, a change that persists in the maternal gut through the early postpartum period [58, 59; but see 60]. Excess GWG or pre-existing overweight/obesity may amplify or modulate these gut microbiota characteristics [61].

Several studies [30, 31, 38, 39, 42] found that women with overweight/obesity or excessive GWG had higher *Staphylococcus* abundance in the milk than women with normal BMI and normal GWG. *Staphylococcus* is often classified as a “core” bacterial genus in milk [13] and is thought to populate human milk from maternal skin microbiota [62]. However, a high abundance of *Staphylococcus* spp. in the gut is associated with the inflammatory states of obesity [10]–thus, it is plausible that high *Staphylococcus* in milk may arise from similarly high *Staphylococcus* in the gut microbiota of women with higher weight status and excess GWG. Although less frequently observed in this review [30, 32], the observation that overweight, obesity and excessive GWG are correlated with lower *Bifidobacterium* levels in milk may also be explained by weight-associated shifts in the gut microbiome. In the gut microbiome, *Bifidobacterium* carries out a variety of functions, such as improving glucose tolerance and reducing plasma levels of lipopolysaccharides [63]. Low abundance of *Bifidobacterium* in the gut microbiome is linked to the low-grade inflammation, gut dysbiosis (including the proliferation of "energy-extractive" microbial species), and metabolic dysregulation found in obesity [64]. In short, the gut microbiome may be affected by maternal weight status, which in turn, may shape milk microbiome composition.

One possible mechanism is the hypothesized entero-mammary pathway, in which maternal gut microbes travel through circulation and feed into the milk microbiome to subsist on prebiotic human milk oligosaccharides [16–18]. Through this pathway, shifts in maternal gut microbiota related to overweight/obesity or excessive GWG may pass on to the milk microbiome. However, the infant oral cavity, the maternal skin microbiome and the surrounding environment are all other sources that seed the milk microbiome [17], and the maternal gut is likely only a small factor shaping its composition. Additional research could attempt to elucidate the relative contribution of the maternal gut microbiome in driving weight-based differences in milk microbial taxa and diversity.

Second, maternal diet may shape weight-based differences in the milk microbiome, either by directly influencing milk microbiota or by influencing other factors of milk composition (e.g., milk macronutrient profiles, human milk oligosaccharides, etc.). Dietary intake during pregnancy is associated with both maternal BMI and GWG [65] and thus, may explain weight-related variations in the milk microbiome. Previous research has shown that maternal fat and fiber intakes during gestation and lactation are associated with the the macronutrient composition of milk [66], the milk microbiome [34, 51, 67], as well as the maternal gut microbiome [68–70]; these studies suggest that maternal diet may directly or indirectly shape milk microbial communities. Based on our review, it is evident that the current literature assessing maternal weight status-milk microbiome relationships also accounts for maternal dietary intake. For instance, two included studies [34, 40] investigated how maternal diet was related to the composition of the milk microbiome in addition to maternal weight status. Williams and colleagues [34] found that saturated and monounsaturated fatty acid intake were inversely associated with *Corynebacterium* abundance and total carbohydrate intake was inversely associated with Firmicutes abundance. One study [40] employed structural equation models to demonstrate that the effect of maternal BMI on the milk microbiome was partly driven by effects of maternal diet; the models also demonstrated that maternal BMI influenced the milk microbiome directly and indirectly, by affecting other components of milk (e.g., human milk oligosaccharides, lipids, and cytokines). Adherence to dietary patterns, such as the “Western” pattern–high in saturated fats, sugar, and ultra-processed foods and low in fiber–not only increases risk of obesity, but is also associated with gut dysbiosis [71]. One possibility is that “Western”-like diets may similarly impact milk microbiome communities directly or indirectly by influencing other milk compositional components or the maternal gut microbiome [40].

Third, breastfeeding practices, stage of lactation, and delivery mode may also explain maternal weight-related differences in milk microbiome composition. Women with obesity and excessive GWG report shorter breastfeeding durations, due to various cultural, psychosocial and physiological factors [72]. In addition, it has been shown that the duration of breastfeeding predicts milk macronutrient composition [73]. Following this logic, shorter durations or less exclusivity of breastfeeding in women with overweight or obesity may alter the composition of milk microbiome (or other milk compositional factors) by affecting how long or how frequently the breast is exposed to the infant oral cavity and resident oral microbes, as well as the skin microbiota around the areola [40]. Indeed, in one study in this review [48], prepregnancy BMI and GWG interacted with breastfeeding status to influence the milk microbiome. Specifically, prepregnancy BMI was associated with overall milk microbiome composition only in exclusively breastfeeding women, but not in mixed-feeding women. Similarly, breastfeeding status only influenced the milk microbiome in women with normal GWG, and not in women with excessive GWG [48]. Along with other studies in this review, these results suggest that breastfeeding patterns and maternal weight status influence the milk microbiome independently and in conjunction with each other. Thus, weight-related differences in milk composition may be partly driven by breastfeeding practices.

Like other components of milk composition, the milk microbiome undergoes transitional changes during the period of lactation; thus, stage of lactation may drive the findings we observe in this review. For instance, one study found that over the course of lactation, there were increases in total bacterial concentration in milk, and to a lesser extent, increasing abundances of specific genera, such as *Bifidobacterium*, *Staphylococcus* and *Lactobacillus* [74]. Four studies [33, 36, 40, 48] reported that stage of lactation was associated with milk microbiome composition, while one study explicitly controlled for stage of lactation by collecting all samples at the same postpartum time point [38]. Stage of lactation, if not accounted for, may confound effects of maternal weight status on milk microbiome composition.

Moreover, women with obesity are more likely to have Caesarian sections [75] and thus, delivery mode may partly explain weight-related differences in the milk microbiome. Infants born via Caesarian section have greater abundances of skin microbes, such as *Staphylococcus*, across various body sites, including the infant oral cavity [76, 77], a site that is known to influence the milk microbiome [40]. However, three of the reviewed studies [41, 42, 48] accounted for delivery mode as a confounder and still observed associations between maternal weight status and milk microbiome composition. Thus, this brings into question whether delivery mode is a strong driver of weight-based variation in the milk microbiome.

### Directions for future research

We offer suggestions for future research to help clarify the relationship between maternal weight status and the milk microbiome. First, many investigations identified in our review relied on BMI, a convenient but sometimes inadequate proxy for maternal nutritional status [62]. Maternal adiposity can be more accurately and precisely measured using skinfolds or dual energy x-ray absorptiometry (DXA). Additional research could employ these techniques to evaluate whether maternal body composition, in conjunction with maternal diet and glucose tolerance status [42], are associated with milk microbiome. Second, the association between maternal weight status and the milk microbiome may be confounded with maternal diet and breastfeeding behavior. We suggest that future research attempt to tease apart these relationships by collecting detailed and triangulated measures of maternal diet (e.g., 24-hour recalls, Food Frequency Questionnaires), breastfeeding behavior (e.g., frequency of breastfeeding), alongside maternal anthropometrics and milk microbiome samples. Third, it remains unclear whether the milk microbiota has clinically and biologically meaningful impacts on intergenerational cycles of obesity. Although preliminary evidence suggests that the milk microbiome colonizes the infant gut to a measurable degree [14, 17], it is not known whether variation in milk microbiome composition (stemming from weight status or other maternal factors) begets variation in infant metabolic outcomes, such as in growth patterns or infant adipose deposition [78]. In fact, whether the milk microbiome independently impacts infant health and development has remained an unanswered question for some time [40, 62]. Future research could address this possibility, for example, by employing statistical models to assess whether milk microbiome variation predicts variation in infant gut microbiota and growth. Fifth, the populations included in this review are from industrialized, high-income, and urbanized countries, and likely do not represent the full scope of global variation in the human milk microbiome and its relationship with weight status. Although some cross-geographic research has been conducted on the human milk microbiome [79], future researchers could investigate how relationships between maternal weight status (using non-BMI measures; [80]) and the milk microbiome vary across populations exposed to different nutritional environments.

### Strengths and limitations

The strengths of this review include the adherence to an *a priori* protocol (registration #: CRD42020165633) and the breadth of the guiding research question, allowing us to identify a broad overview of an area of literature that is still underexplored. The findings of this scoping review can be used to guide research on the relationship between maternal metabolic states, the milk microbiome, and intergenerational risk of obesity. This scoping review also has limitations that warrant consideration. First, included studies differed in many ways including: their methods of milk collection, DNA extraction and amplification, sequencing depth, bioinformatic approaches, participant characteristics (e.g., maternal age, mode of breastfeeding), infant characteristics (e.g. gestational age, birth mode, age at data collection), and in the restrictiveness of inclusion/exclusion criteria. In particular, the hypervariable regions that were sequenced via 16S rRNA sequencing varied substantially between studies. This prevents side-by-side comparisons of results and thus limits the conclusions that can be drawn through analysis. In particular, the studies conducted by Asbury et al. [35, 41] had study objectives focused solely on pre-term infants (<37 weeks gestation) in their sample participants. Gestational age is a known influence on milk microbiome composition and thus caution should be taken when comparing results between pre-term and full-term infants [74, 81]. Second, there may have been publication bias towards statistically significant results in the studies we reviewed—for example, excluded studies may have not reported statistically insignificant relationships between maternal weight status measures and milk microbiome composition. Third, as mentioned above, the populations represented by these extracted studies are mostly from industrialized and urbanized regions of the world. Therefore, with some exceptions, the included studies likely do not represent global variation in the milk microbiome as they are confined to settings within industrialized populations.

## Conclusion

This scoping review examines how measures of maternal weight status associate with the milk microbiome. Using a scoping review methodology, we found that current research supports the claim that maternal pre-pregnancy BMI, postpartum BMI and GWG are associated with distinct compositions of the milk microbiome. We posit that maternal gut dysbiosis and metabolic dysregulation associated with overweight or obesity, as well as other interrelated maternal factors (e.g., maternal diet, breastfeeding practices, delivery mode, and stage of lactation) are intertwined with weight status, and may explain weight-related differences in composition of the milk microbiome. However, additional research is needed to determine whether these maternal weight-related differences in milk microbiota meaningfully impact infant health, gut microbiome development, and later disease risk.

## Supporting information

S1 Table “Database and platform information”. (DOCX)

S2 Table “Search details and strategies”. (DOCX)

S3 Table “Full data extraction”. (DOCX)
