# Supplementary material for: Maternal weight status and the composition of the human milk microbiome: A scoping review
Source: PLoS One. 2022 Oct 3;17(10):e0274950. doi: 10.1371/journal.pone.0274950 (PMC9529148; doi:10.1371/journal.pone.0274950)
Supplement: S1 Table — (DOCX) [file pone.0274950.s001.docx]

# Supplemental Table 1: Database and Platform Information

| Database | Platform and notes |
| --- | --- |
| MEDLINE ALL | Ovid |
| Embase | Ovid |
| CINAHL Complete | EBSCOhost |
| Web of Science Core Collection, as licensed at Yale University | Science Citation Index Expanded (SCI-EXPANDED) --1900-present  Social Sciences Citation Index (SSCI) --1900-present  Arts & Humanities Citation Index (A&HCI) --1975-present  Conference Proceedings Citation Index- Science (CPCI-S) --1991-present  Conference Proceedings Citation Index- Social Science & Humanities (CPCI-SSH) --1991-present  Book Citation Index– Science (BKCI-S) --2005-present  Book Citation Index– Social Sciences & Humanities (BKCI-SSH) --2005-present  Emerging Sources Citation Index (ESCI) --2015-present |
| Scopus | Not applicable |
