# Supplementary material for: Maternal weight status and the composition of the human milk microbiome: A scoping review
Source: PLoS One. 2022 Oct 3;17(10):e0274950. doi: 10.1371/journal.pone.0274950 (PMC9529148; doi:10.1371/journal.pone.0274950)
Supplement: S2 Table — (DOCX) [file pone.0274950.s002.docx]

# Supplemental Tables 2 (A-F): Search Details and Strategies

# Supplemental Table 2a (S2A): Peer-Reviewed Medline Search Strategy

Ovid MEDLINE(R) ALL <1946 to February 13, 2020>

| 2 | [medline -- use medall in http://access.ovid.com/demo/ovidsptools/launcher/dlp_launcher.html] | 0 |
| --- | --- | --- |
| 3 | [-----concept 1 breastmilk] | 0 |
| 4 | milk, human/ | 18919 |
| 5 | breast milk expression/ | 287 |
| 6 | breast feeding/ | 36798 |
| 7 | (breastmilk or breast milk or human milk).mp. | 23174 |
| 8 | (mother* adj2 milk).mp. | 2865 |
| 9 | ((wom#n* or collect* or express* or maternal*) adj1 milk).mp. | 2749 |
| 10 | (lactation or lactating or breastfeed* or breast feed or breast feeds or breast feeding or breastfed or breast fed).mp. | 111438 |
| 11 | [------concept 2 microbiome] | 0 |
| 12 | Milk, Human/mi | 1047 |
| 13 | RNA, Ribosomal, 16S/ | 49016 |
| 14 | (16S adj2 (rRNA or ribosom*)).mp. | 68058 |
| 15 | exp Sequence Analysis, DNA/ | 225763 |
| 16 | (sequenc* adj3 (analys* or analyz* or dna or typing or genom*)).mp. | 511037 |
| 17 | DNA, Bacterial/ | 111427 |
| 18 | exp bacteria/ | 1343726 |
| 19 | (bacteria or bacterial or bacterium or flora or microflora or micro organism* or microorganism*).mp. | 1446868 |
| 20 | microbiota/ | 15106 |
| 21 | microbiology.fs. | 750245 |
| 22 | (microbiome* or microbial or microbiota or microbe or microbes).mp. | 437883 |
| 23 | (next generation sequencing or next gen sequencing or high throughput sequencing).mp. | 44281 |
| 24 | exp High-Throughput Nucleotide Sequencing/ | 29286 |
| 25 | (metaxonomics or metagenomics or ngs or hts).mp. | 25758 |
| 26 | (polymerase chain reaction or pcr or qPCR).mp. | 824228 |
| 27 | exp polymerase chain reaction/ | 444893 |
| 28 | (gel adj1 electrophoresis).mp. | 132220 |
| 29 | Sanger sequencing.mp. | 9113 |
| 30 | [------concept 3 maternal BMI] | 0 |
| 31 | exp "body weights and measures"/ | 596548 |
| 32 | exp body composition/ | 53313 |
| 33 | exp adipose tissue/ | 95585 |
| 34 | (obes* or overweight or weight* or body fat or BMI or body mass or body composition* or adipos* or fatty tissue* or quetelet* or waist*).mp. | 1715908 |
| 35 | [-----exclusions] | 0 |
| 36 | (animals not humans).sh. [exclude this] | 4640750 |
| 37 | english.lg. [limit to this] | 2.6E+07 |
| 38 | [-----experiments and summations] | 0 |
| 39 | or/4-10 [breastmilk concept] | 126588 |
| 40 | or/12-29 [microbiome concept] | 3331020 |
| 41 | or/31-34 [BMI concept] | 1801223 |
| 42 | 39 and 40 and 41 | 1799 |
| 43 | (42 and 37) not 36 | 915 |

# Supplemental Table 2b (S2B): Medline Search Strategy, 2022 Update

Ovid MEDLINE(R) ALL <1946 to February 23, 2022>

| 2 | [medline -- use medall in http://access.ovid.com/demo/ovidsptools/launcher/dlp_launcher.html] | 0 |
| --- | --- | --- |
| 3 | [-----concept 1 breastmilk] | 0 |
| 4 | milk, human/ | 21268 |
| 5 | breast milk expression/ | 376 |
| 6 | breast feeding/ | 41313 |
| 7 | (breastmilk or breast milk or human milk).mp. | 26562 |
| 8 | (mother* adj2 milk).mp. | 3273 |
| 9 | ((wom#n* or collect* or express* or maternal*) adj1 milk).mp. | 3183 |
| 10 | (lactation or lactating or breastfeed* or breast feed or breast feeds or breast feeding or breastfed or breast fed).mp. | 123599 |
| 11 | [------concept 2 microbiome] | 0 |
| 12 | Milk, Human/mi | 1189 |
| 13 | RNA, Ribosomal, 16S/ | 59305 |
| 14 | (16S adj2 (rRNA or ribosom*)).mp. | 83226 |
| 15 | exp Sequence Analysis, DNA/ | 249092 |
| 16 | (sequenc* adj3 (analys* or analyz* or dna or typing or genom*)).mp. | 571488 |
| 17 | DNA, Bacterial/ | 117350 |
| 18 | exp bacteria/ | 1459540 |
| 19 | (bacteria or bacterial or bacterium or flora or microflora or micro organism* or microorganism*).mp. | 1610915 |
| 20 | microbiota/ | 26770 |
| 21 | microbiology.fs. | 812761 |
| 22 | (microbiome* or microbial or microbiota or microbe or microbes).mp. | 529952 |
| 23 | (next generation sequencing or next gen sequencing or high throughput sequencing).mp. | 64716 |
| 24 | exp High-Throughput Nucleotide Sequencing/ | 46242 |
| 25 | (metaxonomics or metagenomics or ngs or hts).mp. | 37479 |
| 26 | (polymerase chain reaction or pcr or qPCR).mp. | 929937 |
| 27 | exp polymerase chain reaction/ | 461581 |
| 28 | (gel adj1 electrophoresis).mp. | 135579 |
| 29 | Sanger sequencing.mp. | 13263 |
| 30 | [------concept 3 maternal BMI] | 0 |
| 31 | exp "body weights and measures"/ | 660078 |
| 32 | exp body composition/ | 60759 |
| 33 | exp adipose tissue/ | 106159 |
| 34 | (obes* or overweight or weight* or body fat or BMI or body mass or body composition* or adipos* or fatty tissue* or quetelet* or waist*).mp. | 1934677 |
| 35 | [-----exclusions] | 0 |
| 36 | (animals not humans).sh. [exclude this] | 4929283 |
| 37 | english.lg. [limit to this] | 28970570 |
| 38 | [-----experiments and summations] | 0 |
| 39 | or/4-10 [breastmilk concept] | 140369 |
| 40 | or/12-29 [microbiome concept] | 3711290 |
| 41 | or/31-34 [BMI concept] | 2028678 |
| 42 | 39 and 40 and 41 | 2183 |
| 43 | (42 and 37) not 36 | 1160 |

# Supplemental Table 2c (S2C): Embase

Embase <1974 to 2022 February 23>

| 2 | [-----concept 1 breastmilk] | 0 |
| --- | --- | --- |
| 3 | exp breast milk/ | 30666 |
| 4 | breast milk expression/ | 452 |
| 5 | exp breast feeding/ | 59258 |
| 6 | (breastmilk or breast milk or human milk).mp. | 40594 |
| 7 | (mother* adj2 milk).mp. | 4017 |
| 8 | ((wom#n* or collect* or express* or maternal*) adj1 milk).mp. | 3740 |
| 9 | (lactation or lactating or breastfeed* or breast feed or breast feeds or breast feeding or breastfed or breast fed).mp. | 142601 |
| 10 | [------concept 2 microbiome] | 0 |
| 11 | RNA 16S/ | 79866 |
| 12 | (16S adj2 (rRNA or ribosom*)).mp. | 73994 |
| 13 | DNA sequence/ | 210431 |
| 14 | (sequenc* adj3 (analys* or analyz* or dna or typing or genom*)).mp. | 638240 |
| 15 | bacterial DNA/ | 56195 |
| 16 | exp bacterium/ | 1708914 |
| 17 | (bacteria or bacterial or bacterium or flora or microflora or micro organism* or microorganism*).mp. | 1779276 |
| 18 | exp microbiome/ | 26161 |
| 19 | (microbiome* or microbial or microbiota or microbe or microbes).mp. | 411336 |
| 20 | (next generation sequencing or next gen sequencing or high throughput sequencing).mp. | 130204 |
| 21 | high throughput sequencing/ | 61380 |
| 22 | (metaxonomics or metagenomics or ngs or hts).mp. | 68175 |
| 23 | (polymerase chain reaction or pcr or qPCR).mp. | 1380714 |
| 24 | exp polymerase chain reaction/ | 1065043 |
| 25 | (gel adj1 electrophoresis).mp. | 257185 |
| 26 | Sanger sequencing.mp. | 38956 |
| 27 | [------concept 3 maternal BMI] | 0 |
| 28 | exp body weight/ | 565157 |
| 29 | exp body composition/ | 113688 |
| 30 | exp adipose tissue/ | 180654 |
| 31 | (obes* or overweight or weight* or body fat or BMI or body mass or body composition* or adipos* or fatty tissue* or quetelet* or waist*).mp. | 2797699 |
| 32 | [-----exclusions] | 0 |
| 33 | exp animals/ not exp humans/ [exclude this] | 4907148 |
| 34 | english.lg. [limit to this] | 32456370 |
| 35 | [-----experiments and summations] | 0 |
| 36 | or/3-9 [breastmilk concept] | 162516 |
| 37 | or/11-26 [microbiome concept] | 4223795 |
| 38 | or/28-31 [BMI concept] | 2819691 |
| 39 | 36 and 37 and 38 | 3788 |
| 40 | (39 and 34) not 33 | 2367 |

# Supplemental Table 2d (S2D): CINAHL Complete

Updated February 24 2022

| S20 | S5 AND S14 AND S19 | 439 |
| --- | --- | --- |
| S19 | S15 OR S16 OR S17 OR S18 |  |
| S18 | (MH "Adipose Tissue+") |  |
| S17 | (MH "Body Composition+") |  |
| S16 | (MH "Body Weights and Measures+") |  |
| S15 | obes* or overweight or weight* or "body fat" or BMI or "body mass" or body-composition* or adipos* or fatty-tissue* or quetelet* or waist* |  |
| S14 | S6 OR S7 OR S8 OR S9 OR S10 OR S11 OR S12 OR S13 |  |
| S13 | (MH "Polymerase Chain Reaction+") |  |
| S12 | (MH "High-Throughput Screening Assays") |  |
| S11 | MW "mi" |  |
| S10 | (MH "Microbiota") |  |
| S9 | (MH "Bacteria+") |  |
| S8 | (MH "Sequence Analysis+") |  |
| S7 | (MH "Milk, Human+/MI") |  |
| S6 | (16S N/2 (rRNA or ribosom*)) or (sequenc* N/3 (analys* or analyz* or dna or typing or genom*)) or bacteria or bacterial or bacterium or flora or microflora or micro-organism* or microorganism* or microbiome* or microbial or microbiota or microbe or microbes or "next generation sequencing" or "next gen sequencing" or "high throughput sequencing" or metaxonomics or metagenomics or ngs or hts or "polymerase chain reaction" or pcr or qPCR or gel NEAR/1 electrophoresis or "sanger sequencing" |  |
| S5 | S1 OR S2 OR S3 OR S4S4 |  |
| S4 | (MH "Breast Feeding") |  |
| S3 | (MH "Milk Expression") |  |
| S2 | (MH "Milk, Human+") |  |
| S1 | (breastmilk or "breast milk" or "human milk" or (mother* N/2 milk) or ((woman or women* or collect* or express* or maternal*) N/1 milk) or lactation or lactating or breastfeed* or "breast feed" or "breast feeds" or "breast feeding" or breastfed or "breast fed") |  |

# Supplemental Table 2e (S2E): Web of Science Core Collection

Updated February 24 2022

| 7 | #5 NOT #6 | 2,887 |
| --- | --- | --- |
| 6 | TI=(rat OR rats OR mouse OR mice OR animal-model*) | 1,840,448 |
| 5 | #1 AND #2 AND #3 and English (Languages) | 3,269 |
| 4 | #1 AND #2 AND #3 | 3,351 |
| 3 | obes* or overweight or weight* or "body fat" or BMI or "body mass" or body-composition* or adipos* or fatty-tissue* or quetelet* or waist* (Topic) | 2,604,807 |
| 2 | (16S NEAR/2 (rRNA or ribosom*)) or (sequenc* NEAR/3 (analys* or analyz* or dna or typing or genom*)) or bacteria or bacterial or bacterium or flora or microflora or micro-organism* or microorganism* or microbiome* or microbial or microbiota or microbe or microbes or "next generation sequencing" or "next gen sequencing" or "high throughput sequencing" or metaxonomics or metagenomics or ngs or hts or "polymerase chain reaction" or pcr or qPCR or gel NEAR/1 electrophoresis (Topic) | 2,756,423 |
| 1 | breastmilk or "breast milk" or "human milk" or (mother* NEAR/2 milk) or ((woman or women* or collect* or express* or maternal*) NEAR/1 milk) or lactation or lactating or breastfeed* or "breast feed" or "breast feeds" or "breast feeding" or breastfed or "breast fed" (Topic) | 271,072 |

# Supplemental Table 2f (S2F): Scopus

Updated February 24 2022

| Query | results |
| --- | --- |
| ( ( TITLE-ABS-KEY ( breastmilk OR "breast milk" OR "human milk" OR ( mother* W/2 milk ) OR ( ( woman OR women* OR collect* OR express* OR maternal* ) W/1 milk ) OR lactation OR lactating OR breastfeed* OR "breast feed" OR "breast feeds" OR "breast feeding" OR breastfed OR "breast fed" ) ) AND ( TITLE-ABS-KEY ( ( 16s W/2 ( rrna OR ribosom* ) ) OR ( sequenc* W/3 ( analys* OR analyz* OR dna OR typing OR genom* ) ) OR bacteria OR bacterial OR bacterium OR flora OR microflora OR micro-organism* OR microorganism* OR microbiome* OR microbial OR microbiota OR microbe OR microbes OR "next generation sequencing" OR "next gen sequencing" OR "high throughput sequencing" OR metaxonomics OR metagenomics OR ngs OR hts OR "polymerase chain reaction" OR pcr OR qpcr OR ( gel W/1 electrophoresis ) OR "sanger sequencing" ) ) AND ( TITLE-ABS-KEY ( obes* OR overweight OR weight* OR "body fat" OR bmi OR "body mass" OR body-composition* OR adipos* OR fatty-tissue* OR quetelet* OR waist* ) ) ) AND NOT ( TITLE ( rat OR rats OR mouse OR mice OR animal-model* ) OR KEY ( rat OR rats OR mouse OR mice OR animal-model* ) ) AND ( LIMIT-TO ( LANGUAGE , "English" ) ) | 2647 |
