# Supplementary material for: Maternal weight status and the composition of the human milk microbiome: A scoping review
Source: PLoS One. 2022 Oct 3;17(10):e0274950. doi: 10.1371/journal.pone.0274950 (PMC9529148; doi:10.1371/journal.pone.0274950)
Supplement: S3 Table — (DOCX) [file pone.0274950.s003.docx]

# Supplemental Table 3: Full data extraction

| **First Author and Year** | **Study design, location, sample size, and maternal weight status measure(s)** | **Sample maternal characteristics** | **Sample infant characteristics** | **Breastfeeding status, method of milk collection** | **Antibiotics, pro/prebiotics** | **Microbiome analysis methods, taxonomic levels examined, confounders adjusted for** | **Method of statistical analysis** |
| --- | --- | --- | --- | --- | --- | --- | --- |
| Cabrera-Rubio et al. 2012 (29) | Longitudinal  Finland  N=18  Prepregnancy weight, prepregnancy BMI, GWG | Milk sampled at 2 days, 1 month and 6 months postpartum (Colostrum, mature milk)  Maternal age = 32 +/- 5.12  Parity: 1 = 33.3%, 2= 33.3%, >2 = 33.3%  Gestational diabetes = 11.1%  Gestational age = 40.40 +/- 1.11  Prepregnancy weight = 76.11  50% vaginal deliveries | 47.4% male infants  Infant weights: birth weight = 3.69 +/- 0.32;  Age 6 months = 8.13 +/- 0.90;  Age 12 months = 9.87 +/- 1.40;  Age 24 months = 12.41 +/- 1.03 | Exclusively breastfed and mixed-fed infants  Duration of exclusive breastfeeding = 4.10 +/- 1.10 months  Manual expression | Maternal antibiotic use: Before delivery = 0%;  During delivery = 16.6%;  After delivery = 5.5% | 16S rRNA sequencing (V1-V2 regions), qPCR  Genus  No adjustment for confounders | Principal components analysis; Pearson's correlation; mixed models; rarefaction curves |
| Collado et al. 2012 (30) | Longitudinal  Finland  N=56  Prepregnancy weight, prepregnancy BMI, GWG | Milk sampled at 1-2 days, 1 month and 6 months postpartum (Colostrum, mature milk)  Maternal age = 30.23 +/- 4.90  Prepregnancy weight = 72.44 +/- 25.30  Parity: 59.0% primiparous  Gestational diabetes = 32.1%  76.8% vaginal deliveries | 46.4% male infants  Infant weights: Birth weight = 3.61 +/- 0.42;  Age 6 months= 8.02 +/- 1.02 | Exclusively breastfed and mixed-fed infants  Manual expression | Maternal antibiotic use: Before delivery = 0%;  During delivery = 14.3%;  After delivery = 10.70% | Quantitative polymerase chain reaction (qPCR)  Genus  No adjustment for confounders | Mann-Whitney U Tests; Chi-Squared tests; mixed models; Spearman's correlation |
| Davé et al., 2016 (31) | Cross-sectional  United States  N=10  Prepregnancy BMI | Milk sampled at 2-4 days postpartum (colostrum)  Maternal age = 25.4 +/- 3.4  Prepregnancy BMI = 27.3 +/- 2.8  100% vaginal deliveries | 50% male infants | Breastfed for at least 6 months  Breast pump | No information | 16S rRNA sequencing (V4 region)  Genus  No adjustment for confounders | Pearson's correlation (r), Principal components analysis |
| Li et al., 2017 (32) | Cross-sectional  Taiwan and mainland China  N=133 (Taiwan = 31  China = 102)  Postpartum BMI | Milk sampled at random time points for each participant (Mean months postpartum: 6.1 +/- 4.0) (colostrum, transitional milk, mature milk)  Maternal age = 28.5 +/- 4.6  Three BMI groups: <18.5 (n=12), 18.5–25.0 (n=87), and >25.0 (n=32)  39% vaginal deliveries | Infant postpartum age = 6.1 +/- 4.0 | Breast pump | No information | 16S rRNA sequencing (V1-V2 region)  Family, Genus  No adjustment for confounders | No information |
| Williams et al., 2017 (33) | Longitudinal  United States  N=21  Prepregnancy BMI, postpartum BMI | Milk sampled at 2, 5, and 10 days, and 1, 2, 3, 4, 5, and 6 months postpartum (Colostrum, transitional milk, mature milk)  Maternal age = 30 +/- 4  Parity = 1.8 +/- 1  Prepregnancy weight = 64 +/- 7 | No information | Breast pump | Excluded mother-infant dyads who took antibiotics from analyses | 16S rRNA sequencing (V1-V3 region)  Phylum, Family, Genus  No adjustment for confounders | Generalized linear mixed models; Spearman rank-order correlation analysis |
| Asbury et al., 2018  (34) | Longitudinal  Canada  N = 30  Prepregnancy BMI | Milk sampled weekly over the first 8 weeks postpartum (colostrum, transitional milk, mature milk) | No information | No information |  | 16S rRNA sequencing (V4 region)  Genus  Adjustment for delivery mode, antibiotic use | Linear and Poisson regressions |
| Li et al., 2017 (35) | Cross-sectional  Guatemala  N = 76  Postpartum BMI | Milk sampled during early (5-46 days) or established (4-6 months) lactation (colostrum, transitional milk, mature milk) | No information | No information |  | 16S rRNA sequencing (region not specified)  Phylum, Family  No adjustment for confounders | No information |
| Boix-Amoros et al., 2019 (36) | Cross-sectional  Spain, Finland, South Africa and China  N=80 (20 per country)  Prepregnancy BMI | Milk sampled at 1 month postpartum (mature milk)  Maternal age = 33.52 +/- 4.87  Prepregnancy BMI = 24.06 +/- 3.85  50% vaginal deliveries | No information | 100% exclusively breastfeeding  Manual expression |  | 18S rRNA sequencing rRNA sequencing (region not specified), 5.8S rRNA sequencing rRNA sequencing (ITS1 region), qPCR, fungal culturing  Phylum, Genus  Adjusted for maternal age, pre-delivery maternal BMI, and antibiotic use at delivery | Linear regression |
| Ding et al., 2019 (37) | Cross-sectional  China  N = 89  Prepregnancy BMI | Milk sampled at 42 days postpartum (mature milk)  Maternal age range = 20-35 years  Prepregnancy BMI, by region: Northeast China = 20.8;  South China = 20.8;  Northwest China = no data;  East China = 21.5  North China = 20.8 | No information | Manual expression, breast pump | No information | Culturing; qPCR; 16S rRNA sequencing (V3-V4 regions)  Genus, Species  No adjustment for confounders | One-way ANOVA; Kruskal-Wallis test; Pearson's correlation |
| Lundgren et al., 2019 (38) | Cross-sectional  United States  N= 155  Prepregnancy BMI, GWG | Milk sampled at 6 weeks (1.5 months) postpartum (mature milk)  Maternal age = 32.4 (20-45)  Parity = 1.8  Gestational diabetes = 5.2% | 47.4% male infants | 70.3% exclusively breastfed, 14.2% combination fed  Manual expression | Maternal antibiotic use: Prenatal period = 14.8%; 4-months postpartum = 11%; Peripartum period = 45.8% | 16S rRNA sequencing (V4-V5 region)  Phylum, Family, Genus, Species  Adjusted for postpartum collection week, gestational weight gain, and antibiotic use (before 4 months postpartum) | Multinomial logistic regression; Linear regression; Kruskal-Wallis rank sum test and Dunn's tests; PERMANOVA |
| Moossavi et al., 2019 (39) | Cross-sectional  Canada  N = 393  Prepregnancy BMI | Milk sampled at 3-4 months postpartum (mature milk)  Maternal age: Ages 20-30 = 25.3%; Ages  30-40 = 68.4%; Ages  >40 = 6.3%  Parity: 0 = 211 (53.7%);  1 = 126; (32.1%);  2+ = 56 (14.2%)  Prepregnancy BMI = 24.3 +/-5.2 | 51.1% male infants  Birth weight (g) = 3469 (SD = 469) | 48.3% exclusively breastfeeding;  41.9% direct breastfeeding (at the breast only)  Manual expression, breast pump | Maternal antibiotic use: Intrapartum = 35.8%; Postpartum period (before 3-4 months) = 10.6%  Infant antibiotic use: Postpartum period (before 3-4 months) = 2.8% | 16S rRNA sequencing (V4 region)  Phylum, Order, Family, Genus, Species  No adjustment for confounders | Multiple linear regression; redundancy analysis; structural equations modeling (confirmatory factor analysis) |
| Asbury et al., 2020 (40) | Longitudinal  Canada  N = 86  Prepregnancy BMI | Milk sampled weekly over the first 8 weeks postpartum (mature milk)  Maternal age = 33.4 +/- 4.8  Prepregnancy BMI = 25.2 +/- 5.5  38% vaginal deliveries | 46% male infants  Birth weight (g) = 879.5 +/- 210.7 | No information  Manual expression, breast pump |  | 16S rRNA sequencing (V4 region)  Genus  Adjusted for postpartum week, gestational age, delivery mode, sequencing batch effects and antibiotic use | Linear mixed effects models; repeated measures Poisson regression models |
| LeMay-Nedjelski et al., 2020 (41) | Cross-sectional  Canada  N = 113  Prepregnancy BMI, postpartum BMI | Milk sampled at 3 months postpartum (mature milk)  Maternal age = 34.2 years +/- 4.2  Gestational diabetes = 21.1%  Prepregnancy BMI = 24.3 +/- 4.6  Postpartum BMI = 26.4 +/- 5.2  56.6% vaginal deliveries | No information | 49.6% Exclusively breastfeeding; 50.4% mixed breastfeeding  Breast pump |  | 16S rRNA sequencing (V4 region) | Multivariable linear regression; multivariable Poisson regression |
| Treven et al., 2019 (42) | Cross-sectional  Slovenia  N = 32  Postpartum BMI | Milk sampled at 3-8 weeks postpartum (transitional milk, mature milk) | No information | No information  Manual expression, breast pump | N=7 women and n=11 infants took probiotics the month before sampling; One woman (n=1) completed antibiotics 2 weeks before sample collection | qPCR; 16S r RNA sequencing (V3-V4 regions); cultivation/matrix-assisted laser desorption/ionization mass spectrometry (MALDI-TOF MS); Sanger sequencing  Phylum, Genus  No adjustment for confounders | Pearson’s correlation; linear discriminant analysis (LefSe) |
| Pace et al., 2021 (43) | Cross-sectional  Ethiopia, The Gambia, Peru, Spain, Sweden, United States  N = 357  Postpartum BMI | Milk sampled at 64.6 +/-21.9 days (mature milk)  Maternal age = 27.4 +/- 6.1  Parity = 2.4 +/- 1.9  Gestational age = 64.6 +/- 21.9  Postpartum BMI = 24.2+/-4.6  86% vaginal deliveries | 50% male infants  Infant weight-for-length z-score = 0.1 +/-0.1 | 60% exclusively breastfeeding  Breast pump | Excluded women and infants who had antibiotics in the 30 days prior sample collection | 16S rRNA sequencing (V1-V3 region)  Genus  No adjustment for confounders | Dirichlet multinomial mixtures modelling (to identify clusters of microbiome types, or “lactotypes”); p Kruskal-Wallis test; Wilcoxon rank test, Chi-squared test; Multiple regression with envfit package in R |
| Bayaga et al., 2021 (44) | Longitudinal  Philippines  N = 34  Postpartum BMI | Milk sampled at 0-4 months postpartum (colostrum, transitional milk, mature milk)  Maternal age = 25.59 +/- 4.71  Parity = 1.41 +/- 1.10  Postpartum BMI: 32.35% overweight, 47.06% normal weight, 20.59% underweight | No information | 100% exclusive breastfeeding  Manual expression | Excluded participants who took probiotics | Culturing | Multiple linear regression; Chi-squared test |
| Yan et al., 2021 (45) | Cross-sectional  China  N = 25  Postpartum BMI | Milk sampled at 7-720 days postpartum (transitional milk, mature milk)  Postpartum BMI: 64% normal weight; 16% overweight: 12% obese; 4% severe obesity  100% vaginal deliveries | 60% male infants | 36% exclusive breastfeeding; 64% partial feeding  Breast pump | Excluded women and infants who took antibiotics (during pregnancy, intrapartum or postnatal periods) | 16S rRNA sequencing (V4-V5 region)  Phylum, Family  No adjustment for confounders | Spearman’s correlation; PERMANOVA |
| Butts et al., 2020 (46) | Cross-sectional  New Zealand  N = 78 | Milk sampled at 6-8 weeks postpartum (mature milk)  Maternal age = 31 +/- 5  Postpartum BMI = 27+/-5  82.1% vaginal deliveries | Infant weight = 3.6 +/- 0.5 | No information | No information | 16S rRNA sequencing (V3-V4 region)  Phylum, Genus  No adjustment for confounders | Kruskal-Wallis non-parametric analysis of variance (ANOVA) |
| Cortes-Macias et al., 2021 (47) | Cross-sectional | Milk sampled within 30 days postpartum (colostrum, transitional milk)  Maternal age = 34.44 +/-3.79  Gestational age = 40 (39-40)  62.5% vaginal deliveries | 44.1% male infants  Infant weight = 3.32 +/- 0.44 | 81.6% exclusively breastfeeding during 1st month postpartum  Breast pump | Antibiotics during pregnancy = 42 +/- 30.9%; Antibiotics during 1st month postpartum = 11 +/- 8.1% | qPCR; 16S rRNA sequencing (V3-V4 region)  Genus  Adjusted for birth mode, feeding practices at 1 month (Poisson regression models); diversity and richness values were adjusted for total bacterial load | Spearman’s correlation; PERMANOVA; discriminant of Principal Components Analysis (DAPC); Redundancy analysis (RDA); t-test; Mann-Whitney U test; Multivariable Poisson regression models |
| Sanjulian et al., 2021 (48) | Cross-sectional | Milk sampled at 2 weeks to 5 years (transitional milk, mature milk)  Maternal age = 35.46 +/- 4.02  Gestational age = 39.76 +/-1.33  86.21% vaginal deliveries | 45.26% male infants | Breast pump | No information | qPCR; 16S rRNA sequencing (V2, V3, V4, V6–7, V8, and V9 regions)  Phylum, Genus  No adjustment for confounders | Pearson’s correlation |

**S3 Legend.** Abbreviations: GWG = Gestational weight gain; EGWG = Excess gestational weight G=gain; OW = Overweight; OB = Obese, qPCR = quantitative polymerase chain reaction, PERMANOVA = permutational analysis of variance; ASV = Amplicon sequence variant, IRR = incidence rate ratio, OR = odds ratio. Gestational age is in weeks, parity indicates number of births, maternal age is in years, infant weight is in kg, BMI is in kg/m^2^, and GWG/EGWG is in kg, unless otherwise noted. All studies followed Institute of Medicine guidelines (6) to categorize GWG (e.g., excessive vs. normal GWG). All values are mean +/- standard deviation, unless otherwise indicated. Information is presented only if it was reported in the original study.
